# Photon upconversion through triplet exciton-mediated energy relay

**DOI:** 10.1038/s41467-021-23967-3

**Published:** 2021-06-17

**Authors:** Sanyang Han, Zhigao Yi, Jiangbin Zhang, Qifei Gu, Liangliang Liang, Xian Qin, Jiahui Xu, Yiming Wu, Hui Xu, Akshay Rao, Xiaogang Liu

**Affiliations:** 1 Department of Chemistry, National University of Singapore, Singapore, Singapore; 2 Department of Physics, Cavendish Laboratory, University of Cambridge, Cambridge, UK; 3 College of Advanced Interdisciplinary Studies, National University of Defense Technology, Changsha, China; 4 Key Laboratory of Functional Inorganic Material Chemistry, Ministry of Education, School of Chemistry and Material Science, Heilongjiang University, Harbin, China; 5 Joint School of National University of Singapore and Tianjin University, International Campus of Tianjin University, Fuzhou, China; 6 Center for Functional Materials, National University of Singapore Suzhou Research Institute, Suzhou, China

**Keywords:** Energy transfer, Nanoparticles, Organic-inorganic nanostructures

## Abstract

Exploration of upconversion luminescence from lanthanide emitters through energy migration has profound implications for fundamental research and technology development. However, energy migration-mediated upconversion requires stringent experimental conditions, such as high power excitation and special migratory ions in the host lattice, imposing selection constraints on lanthanide emitters. Here we demonstrate photon upconversion of diverse lanthanide emitters by harnessing triplet exciton-mediated energy relay. Compared with gadolinium-based systems, this energy relay is less dependent on excitation power and enhances the emission intensity of Tb^3+^ by 158-fold. Mechanistic investigations reveal that emission enhancement is attributable to strong coupling between lanthanides and surface molecules, which enables fast triplet generation (<100 ps) and subsequent near-unity triplet transfer efficiency from surface ligands to lanthanides. Moreover, the energy relay approach supports long-distance energy transfer and allows upconversion modulation in microstructures. These findings enhance fundamental understanding of energy transfer at molecule-nanoparticle interfaces and open exciting avenues for developing hybrid, high-performance optical materials.

## Abstract (web summary)

Photon upconversion in lanthanide-doped nanoparticles enables important technological developments. Here the authors demonstrate a mechanism leading to enhanced upconversion emission in core-shell nanoparticles, and long-distance energy transfer between nanoparticles, through triplet state population of an organic surface ligand.

## Introduction

Optical spectroscopy has witnessed a rapid development of lanthanide-doped nanoparticle probes over the past decade owing to their outstanding features, including sharp emission bandwidths, large anti-Stokes shifts, and high photostability, as well as the ability to convert near-infrared (NIR) excitation to short-wavelength emission^1–4^. These nanoparticles hold great promise in optical imaging^5–9^, sensing^10–14^, optogenetics^15–17^, lasing^18,19^, anticounterfeiting^20,21^, volumetric display^22^, and therapeutics^23–26^. Despite enticing prospects, photon upconversion has been largely limited to a small set of lanthanide activators (e.g., Er^3+^, Tm^3+^, and Ho^3+^) with ladder-like energy levels^27–32^. To this end, considerable effort has been devoted to strategies for lanthanide emitters without long-lived intermediate energy levels^33–37^. Specifically, upconversion luminescence from Tb^3+^ or Eu^3+^ ions has been achieved through cooperative sensitization upconversion^38–41^ or dimerization sensitization^42,43^. However, these upconversion processes often suffer from low conversion efficiency and weak emission. Alternatively, energy migration-mediated upconversion has proven effective for activators without long-lived intermediate energy states. Despite these successes, energy migration upconversion requires a special host lattice, such as Gd^3+^, capable of migrating excitation energy^33^. Moreover, Gd^3+^ excitation through a five-photon process via a Yb^3+^–Tm^3+^ pair requires a relatively high power density, and the excitation energy has a limited diffusion distance, imposing constraints for photon upconversion in large particles.

**Fig. 1:**
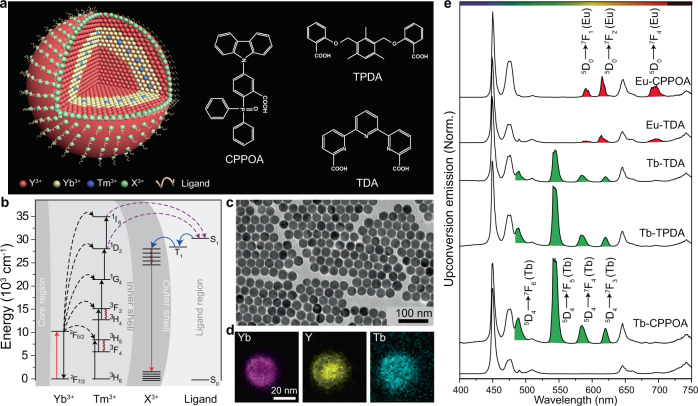
Photon upconversion through triplet exciton-mediated energy relay. **a** Nanoparticle design (NaYF_4_@NaYbF_4_:Tm@NaYF_4_:Tb) and ligand molecules used for nanoparticle conjugation. X^3+^ refers to lanthanide emitters. **b** The proposed energy transfer mechanism underlying photon upconversion in ligand-modified nanoparticles. Note that core, inner shell, outer shell, and surface region are highlighted with different background colours. Yb^3+^ ions first harvest the excitation light and then populate Tm^3+^ ions. The energy transfer from the high-lying excited states of Tm^3+^ ions to singlet states (S_1_) of surface ligand via a Förster resonance energy transfer. Subsequently, triplet states (T_1_) are formed through intersystem crossing. Finally, the excited energy is transferred to lanthanide emitters (X^3+^) on the nanoparticle surface. **c** Transmission electron microscopy imaging of as-synthesized nanocrystals. **d** Elemental Yb, Y, and Tb mapping of a selected nanoparticle. **e** Photoluminescence spectra of NaYF_4_@NaYbF_4_:Tm@NaYF_4_:X (X: Tb^3+^ or Eu^3+^) nanoparticles with and without surface ligands (980-nm excitation, 507 W/cm^2^). Tb^3+^ emission peaks are shown in green and Eu^3+^ emission peaks in red.

Here we demonstrate that it is possible to achieve enhanced upconversion emission through triplet exciton-mediated energy relay (Fig. 1a). Excitation energy is harvested and upconverted via energy transfer in the nanoparticle core. Subsequently, emission in the ultraviolet spectral region is absorbed by capping ligands, followed by back-energy transfer to activators in the shell. This process gives rise to the observed upconversion emission (Fig. 1b). In our design, the surface ligand bridges excitation energy transfer from the core to the shell layer. A suitable organic ligand must satisfy the following criteria: strong broadband absorption in the ultraviolet; a good match between its lower-lying excited states and the activator’s emitting states for efficient back-energy transfer; robust surface coordination and high photostability.

## Results

### Synthesis and characterization

As a proof-of-concept experiment, we chose hexagonal phase NaYF_4_@NaYbF_4_:Tm@NaYF_4_ multilayer, core-shell nanoparticles as a model platform due to their documented high upconversion efficiency^44^. In a typical study, ligand-free core-shell nanoparticles were first synthesized according to a modified literature method^45^. Subsequently, a thin layer of activators (Tb^3+^or Eu^3+^) was coated onto the nanoparticles through cation exchange using TbCl_3_ or EuCl_3_ as the source of activators^46^. Finally, three molecules, namely 9-[3-carboxyl-4-(diphenylphosphinoyl)phenyl]-9H-carbazole (CPPOA), 2,2′-(2,4,6-trimethyl-1,3-phenylene)bis(methylene)bis(oxy)dibenzoic acid (TPDA) and 2,2′:6′,2″-terpyridine-6,6″-dicarboxylic acid (TDA), were conjugated to core-shell particles (Fig. 1a). The absorption of these molecules well matches the emission of Tm^3+^ in the ultraviolet spectral region, whereas their triplet states are close to the emitting states of lanthanide activators (Supplementary Figs. 1 and 2, Supplementary Tables 1–3, and Supplementary Note). Transmission electron microscopic (TEM) imaging shows that the as-synthesized nanoparticles exhibit spherical morphology with an average diameter of 28.7 nm (Fig. 1c and Supplementary Fig. 3). X-ray diffraction confirms the hexagonal phase of the nanocrystals (Supplementary Fig. 4). Compositional analysis of individual nanoparticles by electron energy loss spectroscopy reveals elemental Yb, Y, and Tb distributions within nanoparticles (Fig. 1d). Ligand coordination on nanoparticle surfaces was validated by UV-Vis absorption spectroscopy and Fourier transform infrared spectroscopy (Supplementary Figs. 5 and 6).

### Spectroscopic investigation

We next investigated the luminescence properties of Tb^3+^-exchanged multilayer nanoparticles. Under 980-nm excitation with a continuous-wave (CW) diode laser, ligand-free particles showed blue emission corresponding to ^1^I_6_ → ^3^H_6_ and ^3^F_4_ (290 and 345 nm), ^1^D_2_ → ^3^H_6_ and ^3^F_4_ (360 and 450 nm), ^1^G_4_ → ^3^H_6_ (475 nm), and ^1^G_4_ → ^3^F_4_ (650 nm) optical transitions of Tm^3+^ ions. No Tb^3+^ emission was detected, indicating that no direct energy transfer occurs from Tm^3+^ to Tb^3+^. Upon surface modification with ligands, the particles exhibited a significant decrease in UV emissions. A new set of visible emissions also appeared, arising from ^5^D_4_ → ^7^F_6_ (491 nm), ^7^F_5_ (545 nm), ^7^F_4_ (589 nm), and ^7^F_3_ (622 nm) transitions of Tb^3+^ ions (Fig. 1e and Supplementary Fig. 7). Similarly, by performing a cation-exchange reaction between EuCl_3_ and NaYF_4_@NaYbF_4_:Tm@NaYF_4_ multilayer nanoparticles, we achieved Eu^3+^ emission at 590 nm (^5^D_0_ → ^7^F_1_), 615 nm (^5^D_0_ → ^7^F_2_), and 696 nm (^5^D_0_ → ^7^F_4_). To enrich the color diversity, we doped Eu^3+^ in the outmost NaYF_4_ shell of NaYF_4_@NaYbF_4_:Tm@NaYF_4_ multilayer nanoparticles and then performed a cation-exchange reaction with TbCl_3_. After ligand modification, dual emissions from Tb^3+^ and Eu^3+^ ions were realized. By carefully varying the doping concentration of Eu^3+^, multicolor emission was achieved (Supplementary Fig. 8).

**Fig. 2:**
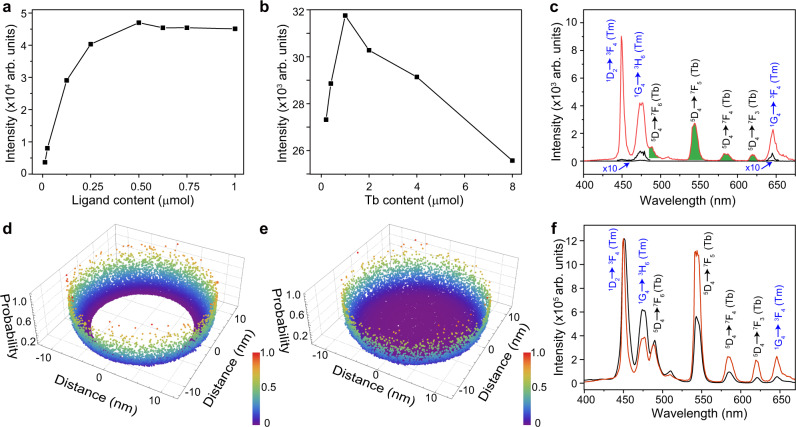
Mechanistic investigation of the energy transfer process. **a** Intensity change in Tb^3+^ emission at 546 nm versus the ligand (CPPOA) content. Samples were excited using a 980-nm laser. **b** Intensity change in Tb^3+^ emission at 546 nm versus the Tb^3+^ content used for cation exchange. Samples were excited using a 980-nm laser. **c** Upconversion luminescence spectra of CPPOA-modified NaYF_4_@NaYbF_4_:1%Tm@NaYF_4_:Tb nanocrystals (red) and NaGdF4:Yb/Tm(49/1 mol%)@NaGdF_4_:Tb (black) obtained by cation exchange under 980-nm excitation (76 W/cm^2^). Note that Tm^3+^ emission (black curve) is enlarged by 10-fold for better visualization. **d**, **e** Monte-Carlo simulation of the excitation energy distribution plotted against the Tm^3+^−surface ligand distance, showing the probability of excitation energy transfer to surface-bound ligand acceptors in a given NaYF_4_@NaYbF_4_:1%Tm@NaYF_4_:Tb multilayer nanoparticle (**d**) and a NaYF_4_:Yb/Tm (30/0.5%)@NaYF_4_:Tb core-shell nanoparticle (**e**). **f** Room-temperature emission spectra of CPPOA-modified NaYF_4_@NaYbF_4_:Tm (1 mol%)@NaYF_4_:Tb (red) and NaYbF_4_:Tm (1 mol%)@NaYF_4_:Tb (black) nanocrystals recorded in ethanol under 980-nm excitation (507 W/cm^2^).

Upconversion emission strongly depends on the number of ligands and lanthanide activators. Here we take CPPOA as an example. In principle, an increased number of CPPOA ligands improves excitation energy harvesting from Yb–Tm pairs in nanoparticles. However, a high loading density of ligands often suffers from concentration quenching, thus dissipating the excitation energy nonradiatively. Similarly, an increase in the activator number generally favors trapping of excitation energy. However, an elevated number of activators can result in deleterious cross-relaxation^47^. By carefully tuning the number of activators and CPPOA molecules, we obtained a maximum emission intensity with 1-μmol Tb^3+^ and 0.5-μmol CPPOA for 15-mg NaYF_4_@NaYbF_4_:Tm@NaYF_4_ multilayer nanoparticles (Fig. 2a, b and Supplementary Figs. 9 and 10; Supplementary Table 4). With optimized concentrations, the Tb^3+^ emission intensity of CPPOA-engineered nanoparticles enhanced 158 times compared with that of a typical Gd^3+^-mediated upconversion nanosystem under a power density of 108 W/cm^2^. The energy-relay nanosystems showed a low power dependence (Supplementary Figs. 11 and 12). When excitation power was further decreased to 76 W/cm^2^, the energy relay-mediated upconversion system showed an intense Tb^3+^ emission, while the energy migration-mediated upconversion system exhibited negligible emission (Fig. 2c). Given that Gd^3+^-based energy migration suffers from luminescence quenching caused by the vibration of surrounding solvent molecules, we tested the optical stability of CPPOA-modified multilayer nanoparticles in various solvents. Surprisingly, these CPPOA-coated nanoparticles showed invariant emission intensity (Supplementary Fig. 13). Moreover, these nanoparticles exhibited high photostability under 980-nm irradiation (Supplementary Fig. 14).

Efficient energy transfer between nanoparticles and surface-bound CPPOA molecules is essential for achieving intense upconversion emission. The core-shell-shell design favors efficient energy transfer from Tm^3+^ donors confined in nanoparticles to surface-bound CPPOA molecules. In this hybrid nanosystem, each Tm^3+^ dopant in the nanoparticle is supposed to be an individual energy donor for transferring excitation energy to all CPPOA acceptors on the particle surface. Thus, overall energy transfer efficiency is determined by averaging all possible distributions of donor–acceptor pairs^48–51^. In the core-shell–shell design, all energy donors (Tm^3+^) are spatially confined in the middle shell. Compared to a random distribution of donors in the core-dominated core-shell structure, this core-shell-shell design may offer higher energy transfer efficiency because of a shorter donor–acceptor distance, as revealed by the Monte-Carlo simulation (Fig. 2d, e). The energy transfer from Tm^3+^ to CPPOA molecules is simulated based on Förster resonance energy transfer due to large separation distances (> 2 nm) between Tm^3+^ and the molecules^52–56^. To validate our hypothesis, we prepared CPPOA-modified NaYbF_4_:Yb/Tm (1 mol%)@NaYF_4_:Tb core-shell nanoparticles as a control. We observed a much higher Tb^3+^ emission intensity from core-shell-shell nanoparticles than from conventional core-shell nanoparticles (Fig. 2f). As an added benefit, the core-shell-shell design enables excitation energy to be confined in the NaYbF_4_ middle shell, which reduces energy loss to crystal lattice defects and increases local excitation energy density. High-density excitation facilitates the accumulation of excitation energy in high-lying states of Tm^3+^ ^44^.

**Fig. 3:**
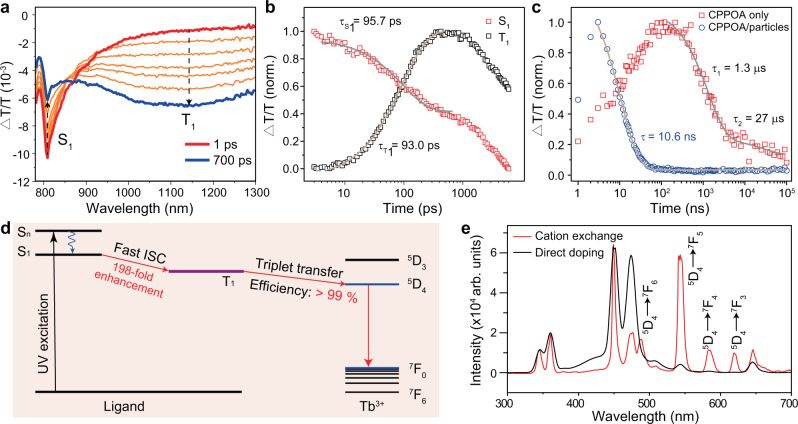
Investigation of energy transfer from ligands to surface emitters. **a** Femtosecond transient absorption spectra of CPPOA-modified NaYF_4_@NaYbF_4_:1%Tm@NaYF_4_:Tb nanoparticles obtained by cation exchange. The excitation wavelength is 345 nm with the pulse energy of 2 μJ. Note that the upward and downward arrows refer to singlet-decay and triplet-rise trends over delay time, respectively. **b** Kinetics of singlet (S_1_) decay and triplet (T_1_) rise, obtained from picosecond transient absorption of the CPPOA-modified nanoparticle solution. **c** Kinetics of triplet decay obtained from nanosecond transient absorption spectra of the CPPOA and CPPOA-modified nanoparticle solution. **d** Simplified energy diagram showing an ultrafast intersystem crossing in CPPOA molecules and triplet energy transfer to Tb^3+^ emitters. **e** Upconversion luminescence spectra of CPPOA-modified NaYF_4_@NaYbF_4_:1%Tm@NaYF_4_:Tb nanoparticles obtained by cation exchange with TbCl_3_ (red) and by direct Tb^3+^-doping (black) under 980-nm excitation (507 W/cm^2^).

Cation-exchange reactions allow a large portion of lanthanide emitters to be exposed at nanoparticle surfaces, enabling direct bonding between surface emitters and capping molecules. Unlike the Tm^3+^-to-CPPOA energy transfer described above, we reasoned that strong exchange coupling dominates energy transfer between molecules and surface lanthanide emitters (e.g., Tb^3+^) because their proximity results in fast triplet generation of molecules and efficient triplet energy transfer from molecules to lanthanide emitters^57^. To validate this hypothesis, we next performed ultrafast transient absorption spectroscopy to probe the photodynamics of excitation energy at the interface of CPPOA and Tb^3+^-exchanged multilayer nanoparticles. The broadband (900−1300 nm) photo-induced absorption feature of molecular triplets appears on a picosecond timescale (Fig. 3a). Kinetics of spectral components reveals a triplet state rise time of 93 ps (Fig. 3b and Supplementary Fig. 15), 198 times faster than that obtained from pristine CPPOA (18.4 ns)^57^. Decay of the T_1_ state has a constant 10.6 ns (Fig. 3c and Supplementary Fig. 16), ~2547-fold faster than that of CPPOA molecules. The quantum efficiency of triplet transfer from CPPOA ligands to nanoparticles was close to 100% (Fig. 3d). To further verify the cation-exchange effect on energy upconversion efficiency, we performed a control experiment by directly doping Tb^3+^ ions into the outmost NaYF_4_ shell of the multilayer nanoparticle. These directly doped nanoparticles showed significantly suppressed Tb^3+^ emission compared to Tb^3+^-exchanged multilayer nanoparticles (Fig. 3e).

### Long-distance energy relay

**Fig. 4:**
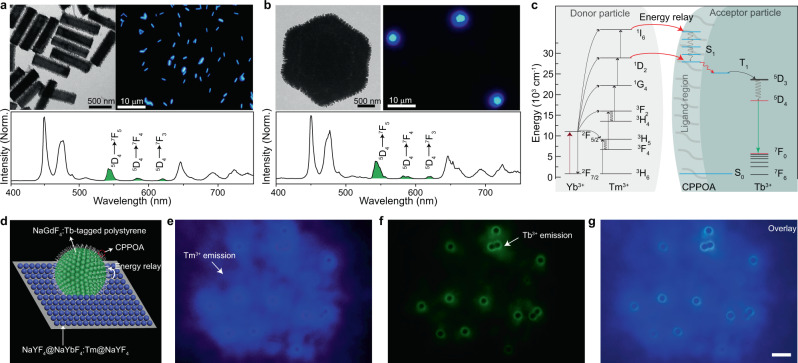
Long-distance energy relay. **a**, **b** Photoluminescence spectra of (**a**) CPPOA-modified NaYF_4_:Yb/Tm (30/0.5 mol%)@NaGdF_4_:Tb microrods and (**b**) NaYbF_4_:Tm (1 mol%)@NaGdF_4_:Tb microplates under 980-nm excitation. Insets show corresponding transmission electron microscopy images (top left) and upconversion microscopic images (top-right). **c** The proposed interparticle energy relay between NaYF_4_@NaYbF_4_:Tm@NaYF_4_ donor particles and NaGdF_4_:Tb accepter nanoparticles. Excitation energies are first harvested and upconverted in donor nanoparticles, and energy transfer then occurs from Tm^3+^ ions in donor nanoparticles to surface ligands (CPPOA) on acceptor nanoparticles. After intersystem crossing in CPPOA molecules, excitation energies are transferred to lanthanide emitters (Tb^3+^) in acceptor nanoparticles, resulting in upconversion emission. **d** Schematic illustration of interparticle energy relay. **e**–**g** Upconversion microscopy images show interparticle energy relay from NaYF_4_@NaYbF_4_:Tm@NaYF_4_ donor particles (blue emission) to polystyrene microspheres tagged with CPPOA-modified NaGdF_4_:Tb nanoparticles (green emission) using a (**e**) 500-nm shortpass filter or (**f**) 540 ± 25 nm bandpass filter. Scale bar: 10 μm.

The large absorption cross-section of the surface ligand provides an opportunity to extract excitation energy from the core over a long distance, achieving multicolor upconversion tuning at a microscopic scale. To test the feasibility of our upconversion process in a microscopic region, we synthesized a set of Tb^3+^-exchanged NaYF_4_:Yb/Tm@NaGdF_4_ hedgehog-like microrods. After coating these microrods with CPPOA molecules, we observed cyan emission under 980-nm excitation (Fig. 4a, top panel), characteristic of Tb^3+^ emission peaks (Fig. 4a, bottom panel). Similarly, we also achieved Tb^3+^ emission from CPPOA-capped NaYF_4_:Yb/Tm@NaGdF_4_:Tb hedgehog-like microplates of 5 μm in diameter (Fig. 4b), which was unattainable by conventional Gd^3+^-mediated energy migration^33^. The long-distance energy relay mediated by surface ligands can also be realized across two sets of nanoparticles. To validate this, we prepared NaYF_4_@NaYbF_4_:Tm@NaYF_4_ nanoparticles as energy donors and NaGdF_4_:Tb/CPPOA-tagged polystyrene microspheres as energy acceptors. Donor particles were first deposited on a glass substrate, and acceptor particles were then drop-casted on the donor particle layer. Upon 980-nm excitation, we observed bright green emission from NaYF_4_:Tb/CPPOA-tagged polystyrene microspheres, suggesting the occurrence of an efficient energy relay process (Fig. 4c–g).

## Discussion

In conclusion, we have demonstrated that photon upconversion from lanthanide activators without ladder-like energy levels can be achieved by a triplet exciton-mediated energy relay. A multilayer core-shell design coupled with specially designed molecules enables precise control over energy transfer and efficient energy relay at the nanoparticle–molecule interface. Compared with Gd^3+^-based energy migration systems, energy relay upconversion offers advantages such as a broad selection of host lattices, high emission intensity, less dependence on excitation power, and high stability in various solvents. Moreover, long-distance energy relay allows upconversion emission to be achieved in microstructures. This development will expand the applicability of lanthanide nanoparticle–molecule hybrid systems for diverse applications.

## Methods

### Materials

Gd(CH_3_CO_2_)_3_•H_2_O, (99.9%), Y(CH_3_CO_2_)_3_•H_2_O (99.9%), Yb(CH_3_CO_2_)_3_•H_2_O (99.9%), Tm(CH_3_CO_2_)_3_•H_2_O (99.9%), Tb(CH_3_CO_2_)_3_•H_2_O (99.9%), Eu(CH_3_CO_2_)_3_•H_2_O (99.9%), oleic acid (90%), 1-octadecene (90%), sodium hydroxide (NaOH; >98%), ammonium fluoride (NH_4_F; >98%), and cyclohexane (anhydrous; >99.5%) were purchased from Sigma-Aldrich. All chemicals were used without further purification.

### Synthesis of NaYF_4_ core nanoparticles

In a typical procedure, an aqueous solution of Y(CH_3_CO_2_)_3_•*x*H_2_O (2.0 mL, 0.2 M) was mixed with 3.0 mL of oleic acid (OA) in a 50-mL flask. The resulting mixture was heated to 150 °C in an oil bath and maintained at this temperature for 30 min, at which time 7.0 mL of 1-octadecene (ODE) were added to the flask. After the mixture was cooled to 50 °C over 30 min, a 6.0-mL methanol solution containing NH_4_F (1.6 mmol) and NaOH (1.0 mmol) was added to the core precursor and stirred for 30 min. After removing volatile solvents, the temperature was increased to 290 °C under an argon atmosphere. After heating for 2 h, the mixture was cooled to room temperature and washed several times with ethanol. Products were re-dispersed in 4.0 mL of cyclohexane.

### Synthesis of NaYF_4_@NaYbF_4_:1%Tm nanoparticles

The NaYbF_4_:1%Tm shell precursor was prepared by mixing an aqueous solution of Ln(CH_3_CO_2_)_3_•*x*H_2_O (2.0 mL, 0.2 M, Ln = Yb or Tm) with 3.0 mL of OA and 7.0 mL of ODE. The mixture was heated at 150 °C in an oil bath for 1 h. After cooling to 80 °C, NaYF_4_ core nanoparticles in 4.0 mL of cyclohexane were added to the mixture and kept at 80 °C for 30 min. Subsequently, a methanol solution of NH_4_F (1.6 mmol) and NaOH (1.0 mmol) was added at 50 °C and stirred for 30 min. After removing volatile solvents, the mixture was heated to 290 °C under an argon atmosphere and maintained at this temperature for 3 h. After cooling to room temperature, the products were precipitated, washed several times with ethanol, and re-dispersed in 4.0 mL of cyclohexane for further use.

### Synthesis of NaYF_4_@NaYbF_4_:1%Tm@NaYF_4_ nanoparticles

The NaYF_4_ shell precursor was prepared by mixing 1.0 mL of Y(CH_3_CO_2_)_3_•*x*H_2_O solution (0.2 M) with 3.0 mL of OA and 7.0 mL of ODE. The mixture was heated at 150 °C in an oil bath for 1 h. After cooling to 80 °C, the as-prepared NaYF_4_@NaYbF_4_:1%Tm nanoparticles were added to the mixture and kept at 80 °C for 30 min. Subsequently, a methanol solution of NH_4_F (0.8 mmol) and NaOH (0.5 mmol) was added under intense agitation at 50 °C. After 30 min, volatile solvents were evaporated by heating at 100 °C for 15 min. The mixture was then heated at 290 °C under an argon atmosphere for 2 h. After cooling to room temperature, nanoparticle products were collected, washed several times with ethanol, and re-dispersed in 4.0 mL of cyclohexane for further use^44^.

### Synthesis of NaYF_4_:Yb/Tm (30/0.5 mol%) microrods

The microrod synthesis was performed according to a hydrothermal method^58^. A mixture of YCl_3_ (1.0 mL, 0.2 M), OA (5.0 mL), ethanol (5.0 mL) and NaOH (0.3 g) was stirred at room temperature. After 20 min, an aqueous solution of NH_4_F (2.0 M, 1.0 mL) was added and kept for another 30 min. The mixture was then transferred to a Teflon-lined autoclave (20 mL) and heated at 220 °C for 12 h. The precipitate was collected, washed several times with ethanol, and re-dispersed in cyclohexane (4.0 mL) for further use.

### Synthesis of 5-μm NaYbF_4_:Tm (1 mol%) microplates

In a typical procedure^58^, a mixture of sodium citrate (0.5 mL; 0.3 M) and YbCl_3_ (2.0 mL; 0.2 M) was stirred at room temperature to form a milky suspension. After that, an aqueous solution of NaF (9.6 mL; 0.5 M) was added. The resulting mixture was then transferred to a Teflon-lined autoclave (20 mL) and heated at 220 °C for 12 h.

### Synthesis of gadolinium trifluoroacetate precursors

In a typical experiment, Gd_2_O_3_ (5.0 mmol) was first dissolved in trifluoroacetic acid (10 mL) at 80 °C, and then dried at 100 °C. The as-prepared lanthanide trifluoroacetate powder was dissolved in water to form gadolinium precursors (0.2 M, 50 mL).

### Synthesis of hierarchical microstructures

This synthesis was performed by an epitaxy growth method^59^. Typically, a cyclohexane dispersion of the as-prepared microrod or microplate seeds (1.0 mL) was first added to a mixture of OA (3.0 mL), ODE (3.0 mL), gadolinium trifluoroacetates (0.2 mmol), and sodium trifluoroacetate (0.36 mmol). After heating to 100 °C and degassing for 30 min, the mixture was heated under a nitrogen atmosphere to 310 °C with a 12 °C/min rate and maintained at that temperature for 30 min. The products were collected, washed several times with ethanol, and re-dispersed in cyclohexane for further use. The same protocol was adopted to synthesize hierarchical NaYbF_4_:Tm (1 mol%)@NaGdF_4_ microplates except for using the microplates as seeds.

### Preparation of ligand-free nanoparticles

The as-prepared hydrophobic nanoparticles were precipitated by the addition of ethanol and then dispersed in an acidic ethanol solution (0.1 M HCl) by ultrasonication. Nanocrystals were collected by centrifugation at 33,481 × *g* for 20 min. The resulting products were washed several times with ethanol and deionized water, and re-dispersed in deionized water.

### Preparation of Tb^3+^-exchanged core-shell-shell nanoparticles

These nanoparticles were prepared by cation-exchange reactions^46^. In a typical synthetic process, the as-prepared ligand-free nanoparticles (500 μL) were mixed with different amounts of TbCl_3_ at room temperature. After 24 h, the nanoparticles were collected by centrifugation at 39,846 × *g* for 30 min and washed several times with ethanol. Finally, the products were re-dispersed in 1.0 mL of ethanol.

### Ligand modification to nanoparticle surfaces

An ethanol solution of ligand-free nanoparticles (1.0 mL) was separated into two equal portions. CPPOA ligands were added to the particle solution and ultrasonicated for 1 h to ensure ligand coordination to nanoparticle surfaces. After that, excess ligands were removed by centrifugation. Finally, the products were re-dispersed in ethanol for optical measurement.

### Preparation of nanoparticle-tagged polystyrene beads

In a typical experiment^33^, an aqueous solution containing ligand-free nanocrystals (50–100 μL, 2 wt%) was mixed with polystyrene bead dispersion (5.0 μL) and butanol (200 μL) at room temperature. After 30 min, the final products were collected by centrifugation at 4427 g for 5 min, washed with H_2_O, and re-dispersed in deionized water.

### Synthesis of 2-(diphenylphosphoryl)-5-bromotoluene (TPPOMBr) molecules

The synthetic procedure is shown in Supplementary Fig. 17. In a typical experiment, 2.97 g (10.0 mmol) of 2-iodo-5-bromotoluene were dissolved in 20.0 mL of anhydrous ether. The solution was added dropwise to a mixture comprising magnesium chips (264 mg; 11.0 mmol) and a small amount of iodine. The mixture was heated at 40 °C for 1 h, and then cooled to 0 °C in an ice bath. A diluted solution of diphenylphosphine chloride (2.65 g; 12.0 mmol) in 10.0 mL of anhydrous ether was added dropwise. After addition, the reaction temperature was increased to room temperature and then stirred overnight. The reaction was quenched by adding 20.0 mL of water and extracted by dichloromethane (3 × 30 mL). The organic layers were dried with anhydride Na_2_SO_4_. The solvent was removed in vacuo. The residue was dissolved using 50 mL of dichloromethane. After the addition of 3-mL hydrogen peroxide (30%), the reaction mixture was stirred for 4 h and extracted using sodium hydrogensulfite-saturated solution. The organic layers were dried with anhydride Na_2_SO_4_. The solvent was removed in vacuo. The product was purified by flash column chromatography to afford a white powder in 80% yield ^1^H-NMR (TMS, DMSO-d_6_, 400 MHz): *δ* = 7.66–7.63 (m, 3H), 7.601–7.56 (m, 8H), 7.508 (d, *J* = 8.4 Hz, 1H), 6.91 (q, *J*_1_ = 8.4 Hz*, J*_2_ = 13.2 Hz, 1H), 2.29 ppm (s, 3H); ^13^C-NMR (TMS, DMSO-d_6_, 100 MHz): *δ* = 145.2, 145.1, 135.0, 134.9, 134.8, 133.2, 132.7, 132.6, 132.2, 131.9, 131.8, 131.5, 130.5, 129.5, 129.4, 129.2, 129.1, 126.8, 126.7; ^31^P-NMR (TMS, DMSO-d_6_, 121.5 MHz): *δ* = 28.6; ESI-MS: *m*/*z* (%): 371 (44) [M+H]^+^.

### Synthesis of 4-(diphenylphosphoryl)-3-methyl-*N*-carbazole (CPPOM) molecules

Under an argon atmosphere, a mixture of TPPOMBr (371 mg; 1.0 mmol), carbazole (501 mg; 3.0 mmol), copper iodide (19 mg; 0.1 mmol), and potassium carbonate (415 mg; 3.0 mmol) was heated to 190 °C and stirred overnight. The reaction was then cooled to room temperature and extracted using water and dichloromethane. The organic layers were dried with anhydride Na_2_SO_4_. The solvent was removed in vacuo. The residue was purified by flash column chromatography to afford a yellowish powder (240 mg, 49% yield) ^1^H-NMR (TMS, DMSO-d_6_, 400 MHz): *δ* = 8.25 (d, *J* = 8.0 Hz, 2H), 7.73–7.60 (m, 11H), 7.56 (q, *J*_1_ = 8.4 Hz*, J*_2_ = 18 Hz, 3H), 7.45 (*t*, *J* = 7.6 Hz, 2H), 7.32–7.21 (m, 3H), 2.43 ppm (s, 3H); ^13^C-NMR (TMS, DMSO-d_6_, 100 MHz): *δ* = 145.2, 145.1, 140.7, 140.7, 140.1, 135.2, 135.1, 133.6, 132.6, 132.0, 131.9, 130.9, 129.8, 129.8, 129.7, 129.6, 129.5, 129.4, 126.9, 123.8, 123.7, 123.5, 121.0, 120.9, 110.4; ^31^P-NMR (TMS, DMSO-d_6_, 121.5 MHz): *δ* = 28.5; ESI-MS: *m*/*z* (%): 458 (84) [M+H]^+^.

### Synthesis of 5-carbazol-9-yl-2-(diphenylphosphinoyl)-benzoic acid (CPPOA) molecules

CPPOM (457 mg; 1.0 mmol) was dissolved in 5.0 mL of pyridine and 2.0 mL of water. The solution was heated to reflux, followed by the addition of potassium permanganate (632 mg; 4.0 mmol). The reaction was refluxed until the purple color disappeared. The reaction was cooled to room temperature and extracted using dichloromethane (3 × 30 mL). The organic layers were dried with anhydride Na_2_SO_4_. The solvent was removed in vacuo. The residue was recrystallized in acetone to yield white crystals (131 mg, 27% yield) ^1^H-NMR (TMS, DMSO-d_6_, 400 MHz): *δ* = 13.40 (s, 1H), 8.27 (d, *J* = 7.6 Hz, 2H), 8.10 (*t*, *J* = 2.4 Hz, 1H), 7.99 (d, *J* = 8.4 Hz, 1H), 7.814 (q, *J*_1_ = 8.4 Hz*, J*_2_ = 13.2 Hz, 1H), 7.71–7.66 (m, 4H), 7.63–7.65 (m, 8H), 7.484 (*t*, *J* = 7.6 Hz, 2H), 7.40 ppm (*t*, *J* = 7.2 Hz, 2H); ^13^C-NMR (TMS, DMSO-d_6_, 100 MHz): *δ* = 167.6, 167.6, 140.7, 140.6, 139.9, 139.3, 139.3, 136.9, 136.8, 134.7, 133.6, 132.1, 132.0, 131.9, 131.8, 131.1, 130.1, 129.0, 128.9, 128.7, 128.6, 127.7, 127.6, 127.0, 123.7, 121.1, 121.1, 110.3; ^31^P-NMR (TMS, DMSO-d_6_, 121.5 MHz): *δ* = 28.8; ESI-MS: *m*/*z* (%): 486 (41) [M−H]^−^.

### Synthesis of 2,2′-(2,4,6-trimethyl-1,3-phenylene)bis(methylene)bis(oxy)dibenzoic acid (TPDA)

TPDA molecules were synthesized according to literature^60^. Anhydrous K_2_CO_3_ (690 mg; 5.0 mmol) was added to an acetone (25.0 mL) solution containing 2,4-bis(bromomethyl)-1,3,5-trimethylbenzene (673 mg, 2.2 mmol), and salicylic acid (761 mg; 5.0 mmol). The resulting mixture was refluxed under magnetic stirring for 8 h. Afterward, water was introduced to the mixture, resulting in a white precipitate. The solid was washed with water before drying. Subsequently, the sample (449 mg; 1.0 mmol) was added to a mixed solution of methanol (25 mL), water (5 mL), and NaOH (120 mg; 3.0 mmol). The reaction mixture was refluxed for 3 h and then filtered, and HCl (1.0 M) was added to the filtrate. The precipitate was filtered and washed with water ^1^H-NMR (500 MHz, DMSO-d_6_) δ = 2.28 (s, 6H), 2.36 (s, 3H), 4.46 (s, 4H), 6.78 (s, 1H), 6.93 (dd, *J* = 9.0, 17.5 Hz, 4H), 7.51 (*t*, *J* = 7.5 Hz, 2H), 7.79 (d, *J* = 7.5 Hz, 2H), 11.27 (br, 2H); ^13^C-NMR (125 MHz, DMSO-d_6_) *δ* = 172.00, 161.20, 136.75, 135.75, 135.71, 135.55, 130.34, 129.36, 119.26, 117.17, 57.55, 19.34, 14.70.

### Synthesis of 2,2′:6′,2″-terpyridine-6,6″-dicarboxylic acid (TDA)

The synthetic procedure for TDA molecules is shown in Supplementary Fig. 18. In a typical procedure^61^, 0.47 g of 2,2′,2″-terpyridine (2.0 mmol), and 20 mL of CH_2_Cl_2_ were added to a solution of 1.30 g of *m*-chloroperbenzoic acid (7.5 mmol) in 20 mL of CH_2_Cl_2_. The mixture was stirred at room temperature for 13 h. After washing with 5% Na_2_CO_3_ solution and drying over anhydrous MgSO_4_, the solvent was evaporated and purified by washing with acetone to give the product (0.44 g, 83% yield). A sample product (2.17 g) was mixed with CH_2_Cl_2_ (80 mL). Then, Me_3_SiCN (12.9 g; 82 mmol) was added to the mixture. After stirring for five min, PhCOCl (3.66 g; 32.8 mmol) was added over 15 min and the mixture was stirred overnight at room temperature. The mixture was neutralized with 10% K_2_CO_3_ solution, and the aqueous phase was extracted with CHCl_3_. The combined organic phase was evaporated and the residue was crystallized from MeCN/THF for further use.

Subsequently, the resulting compound (300 mg) was added to a solution containing ethanol (19.0 mL), water (4.0 mL) and KOH (594 mg). The reaction mixture was refluxed overnight, and then the solvent was evaporated under vacuum. The residue was dissolved in water (5 mL) and the pH was adjusted to 4 with aqueous HCl (1 M). The precipitate was removed by filtration and washed with cold water and acetonitrile. After that, the solid material was heated to reflux in a mixture of H_2_SO_4_/CH_3_COOH (9 mL, 1:1) for 5 h and the reaction mixture was poured onto ice (45 mL). The product was filtered and washed with cold water and acetonitrile to yield the product (TDA, 312 mg, 92% yield) as a white solid ^1^H-NMR (500 MHz, DMSO-d_6_) δ = 8.18 (d, *J* = 6.5 Hz, 2H), 8.21 (*t*, *J* = 7.5 Hz, 3H), 8.65 (d, *J* = 7.5 Hz, 2H), 8.87 (d, *J* = 6.5 Hz, 2H), 13.29 (s, 2H); ^13^C-NMR (125 MHz, DMSO-d_6_) *δ* = 166.44, 155.45, 154.58, 148.52, 139.36, 139.30, 125.53, 124.35, 122.19.

### Monte-Carlo modeling of energy transfer from Tm^3+^ to surface ligands

The energy transfer process is described based on a Förster resonance energy transfer (FRET) mechanism. Monte-Carlo calculations of energy transfer efficiency were executed using Mathematica 12 and performed in a simulated 3D coordinate matrix. In CPPOA-modified nanoparticles, each Tm^3+^ ion can be simplified as a point randomly distributed within a nanoparticle and serve as an individual energy donor. Each CPPOA ligand can be assumed as a point-like energy acceptor randomly distributed on the particle surface. The Monte-Carlo simulation was generated using the following criteria. First, 5000 Tm^3+^ ions are randomly distributed in the core of a core-shell structure or confined within the middle shell of a core-shell-shell structure, while 2000 dye molecules are randomly distributed on particle surfaces. Therefore, every Tm^3+^ ion can transfer the excited energy to all surface-bound dyes. As such, each Tm^3+^-CPPOA pair has a specific separation. The calculated FRET efficiency is the assumption of efficiencies for 2000 energy transfer events, which is defined as the probability of finding Tm^3+^ ions that can transfer the excitation energy to all surface-bound dye molecules. Using the same method, we can derive all the probability of finding 5000 Tm^3+^ donors to offer FRET in a core-shell or core-shell-shell particle. Thus, we can plot the probability of finding energy donors against the spatial distance from the particle surface.

### Quantum mechanical calculations

Density functional theory (DFT) calculations were performed for geometry optimization and harmonic frequency analysis of CPPOA, TPDA and TDA molecules using Gaussian 16 (A.03) program^62^. We chose M06-2X functional in combination with 6-311G** basis set^63,64^. No imaginary frequency was detected for these optimized molecular structures. Time-dependent density functional theory (TD-DFT) calculations were performed to obtain S_1_ and T_1_ energy states of the three molecules. For excited-state calculations, we employed the same functional and basis set as ground-state calculations.

### Characterizations

The structures of as-synthesized molecules were confirmed by nuclear magnetic resonance (NMR) and high-resolution mass spectroscopy ^1^H-NMR, ^13^C-NMR, ^31^P-NMR, and high-resolution mass spectra for the 2-(Diphenylphosphoryl)-5-bromotoluene (TPPOMBr) were shown in Supplementary Figs. 19–22. ^1^H-NMR, ^13^C-NMR, ^31^P-NMR, and high-resolution mass spectra for 4-(diphenylphosphoryl)-3-methyl-*N*-carbazole (CPPOM) were shown in Supplementary Figs. 23–26. ^1^H-NMR, ^13^C-NMR, ^31^P-NMR, and high-resolution mass spectra for 9-[3-carboxyl-4-(diphenylphosphinoyl)phenyl]-9H-carbazole (CPPOA) were shown in Supplementary Figs. 27–30. ^1^H-NMR and ^13^C-NMR for the 2,2′-(2,4,6-trimethyl-1,3-phenylene)bis(methylene)bis(oxy)dibenzoic acid (TPDA) were shown in Supplementary Figs. 31 and 32. ^1^H-NMR, ^13^C-NMR and high-resolution mass spectra for 2,2′:6′,2″-terpyridine-6,6″-dicarboxylic acid (TDA) were shown in Supplementary Figs. 33 and 34.

Powder X-ray diffraction (XRD) data were measured with an X-ray diffractometer (Bruker D8 Advance) with graphite monochromic CuKα radiation (*λ* = 1.5406 Å). Low-resolution transmission electron microscopy (TEM) was performed on a transmission electron microscope (JEOL-1400) operating at an acceleration voltage of 120 kV. High-resolution TEM images were taken using a transmission electron microscope (JEOL-JEM 2010) operated at an acceleration voltage of 200 kV. Energy-dispersive X-ray (EDX) spectroscopic analysis was performed using an energy system (Oxford INCA) operated at 200 kV. UV-Vis spectra were recorded on a UV spectrophotometer (Shimadzu 2450). Fourier transform infrared (FT-IR) spectroscopy employed an FT-IR spectrometer (Varian 3100). Photoluminescence spectra were recorded in a fluorescence spectrophotometer (Edinburgh FSP920) equipped with a photomultiplier (PMT) in conjunction with a 980-nm diode laser and a xenon arc lamp (Xe900). The lanthanide concentration was determined by a Perkin Elmer Avio 500 inductively coupled plasma optical emission spectrometer (ICP-OES).

### Transient absorption spectroscopy

Picosecond time measurements were performed using a commercial transient absorption spectrometer (HELIOS, UltrafastSystems). Pump pulses (355 nm) were generated through a TOPAS-Prime amplifier (Light Conversion), which was pumped by a 790-nm pulse laser from a regenerative Ti:Sapphire amplifier system (Spectra Physics, Solstice). The broadband probe pulse (800–1550 nm) was obtained by introducing a 790-nm laser pulse into a YAG crystal. The pump-probe delay was computer-controlled with a piezoelectric translation stage. The time resolution of laser pulses was approximately 200 fs. Nanosecond time measurements were performed with an electronically controlled delay.

Samples were excited with a pump pulse and probed at different delay times using a broadband probe pulse. A differential transmission (ΔT/T) signal in transient absorption spectra was recorded over short pulses (500 fs to 6 ns) with a probe covering (500–850 nm, 750–1600 nm) and long (1 ns to 1 ms) time delays with a probe pulse covering (350–750 nm, 850–1020 nm). To identify different components from transient absorption data, a genetic algorithm analysis was also used to distinguish different spectral species and corresponding kinetics. Triplet energy transfer efficiency can be calculated by *η* = (*τ*_donor only_ − *τ*_donor–acceptor)_/*τ*_donor only_. Note that *τ*_donor only_ refers to the triplet lifetime of the donor in the absence of acceptors, while *τ*_donor–acceptor_ represents the triplet lifetime of the donor in the presence of acceptors.

## Supplementary information

Supplementary Information

## Data Availability

All data supporting the findings of this study are publicly available from the University of Cambridge repository at 10.17863/CAM.69276.

## Code availability

Codes for Monte-Carlo simulation are publicly available from the University of Cambridge repository at 10.17863/CAM.69276.

## References

[1] Auzel F (2004). Upconversion and anti-Stokes processes with f and d ions in solids. Chem. Rev..

[2] Haase M, Schäfer H (2011). Upconverting nanoparticles. Angew. Chem. Int. Ed..

[3] Bünzli J-CG (2010). Lanthanide luminescence for biomedical analyses and imaging. Chem. Rev..

[4] Gai S, Li C, Yang P, Lin J (2014). Recent progress in rare earth micro/nanocrystals: soft chemical synthesis, luminescent properties, and biomedical applications. Chem. Rev..

[5] Liu Q (2018). Single upconversion nanoparticle imaging at sub-10 W cm^−2^ irradiance. Nat. Photon..

[6] Liu Y (2017). Amplified stimulated emission in upconversion nanoparticles for super-resolution nanoscopy. Nature.

[7] Fan Y (2018). Lifetime-engineered NIR-II nanoparticles unlock multiplexed in vivo imaging. Nat. Nanotechnol..

[8] Gu Y (2019). High-sensitivity imaging of time-domain near-infrared light transducer. Nat. Photon..

[9] Nam SH (2011). Long-term real-time tracking of lanthanide ion doped upconverting nanoparticles in living cells. Angew. Chem. Int. Ed..

[10] Zheng W (2015). Lanthanide-doped upconversion nano-bioprobes: electronic structures, optical properties, and biodetection. Chem. Soc. Rev..

[11] Fischer LH, Harms GS, Wolfbeis OS (2011). Upconverting nanoparticles for nanoscale thermometry. Angew. Chem. Int. Ed..

[12] Vetrone F (2010). Temperature sensing using fluorescent nanothermometers. ACS Nano.

[13] Lay A (2018). Bright, mechanosensitive upconversion with cubic-phase heteroepitaxial core–shell nanoparticles. Nano Lett..

[14] Zhou J, del Rosal B, Jaque D, Uchiyama S, Jin D (2020). Advances and challenges for fluorescence nanothermometry. Nat. Methods.

[15] Chen S (2018). Near-infrared deep brain stimulation via upconversion nanoparticle-mediated optogenetics. Science.

[16] Wu X (2016). Dye-sensitized core/active shell upconversion nanoparticles for optogenetics and bioimaging applications. ACS Nano.

[17] Shah S (2015). Hybrid upconversion nanomaterials for optogenetic neuronal control. Nanoscale.

[18] Fernandez-Bravo A (2018). Continuous-wave upconverting nanoparticle microlasers. Nat. Nanotechnol..

[19] Jin L, Chen X, Siu C, Wang F, Yu S (2017). Enhancing multiphoton upconversion from NaYF_4_:Yb/Tm@NaYF_4_ core–shell nanoparticles via the use of laser cavity. ACS Nano.

[20] Lu Y (2014). Tunable lifetime multiplexing using luminescent nanocrystals. Nat. Photon..

[21] Lee J (2014). Universal process-inert encoding architecture for polymer microparticles. Nat. Mater..

[22] Deng R (2015). Temporal full-colour tuning through non-steady-state upconversion. Nat. Nanotechnol..

[23] Hou Z (2019). Hydrogenated titanium oxide decorated upconversion nanoparticles: facile laser modified synthesis and 808 nm near-infrared light triggered phototherapy. Chem. Mater..

[24] Chen G, Qiu H, Prasad PN, Chen X (2014). Upconversion nanoparticles: design, nanochemistry, and applications in theranostics. Chem. Rev..

[25] Chan M‐H (2017). Minimizing the heat effect of photodynamic therapy based on inorganic nanocomposites mediated by 808 nm near-infrared light. Small.

[26] Song R (2020). Near‐infrared light‐triggered chlorine radical (.Cl) stress for cancer therapy. Angew. Chem. Int. Ed..

[27] Dong H, Sun L, Yan C (2015). Energy transfer in lanthanide upconversion studies for extended optical applications. Chem. Soc. Rev..

[28] Zhuo Z (2017). Manipulating energy transfer in lanthanide-doped single nanoparticles for highly enhanced upconverting luminescence. Chem. Sci..

[29] Lu D (2020). Exploring single-nanoparticle dynamics at high temperature by optical tweezers. Nano Lett..

[30] Zou J (2018). Precisely tailoring upconversion dynamics via energy migration in core–shell nanostructures. Angew. Chem. Int. Ed..

[31] Vetrone F, Boyer J-C, Capobianco JA, Speghini A, Bettinelli M (2004). Significance of Yb^3+^ concentration on the upconversion mechanisms in codoped Y_2_O_3_: Er^3+^, Yb^3+^ nanocrystals. J. Appl. Phys..

[32] Wang Z, Meijerink A (2018). Concentration quenching in upconversion nanocrystals. J. Phys. Chem. C.

[33] Wang F (2011). Tuning upconversion through energy migration in core-shell nanoparticles. Nat. Mater..

[34] Dong H (2017). Versatile spectral and lifetime multiplexing nanoplatform with excitation orthogonalized upconversion luminescence. ACS Nano.

[35] Ledoux G (2018). Modeling energy migration for upconversion materials. J. Phys. Chem. C.

[36] Tu L, Liu X, Wu F, Zhang H (2015). Excitation energy migration dynamics in upconversion nanomaterials. Chem. Soc. Rev..

[37] Zhou B (2020). NIR II-responsive photon upconversion through energy migration in an ytterbium sublattice. Nat. Photon..

[38] Prorok K (2014). The impact of shell host (NaYF_4_/CaF_2_) and shell deposition methods on the up-conversion enhancement in Tb^3+^, Yb^3+^ codoped colloidal α-NaYF_4_ core–shell nanoparticles. Nanoscale.

[39] Prorok K, Pawlyta M, Stręk W, Bednarkiewicz A (2016). Energy migration up-conversion of Tb^3+^ in Yb^3+^ and Nd^3+^ codoped active-core/active-shell colloidal nanoparticles. Chem. Mater..

[40] Xue M (2016). Highly enhanced cooperative upconversion luminescence through energy transfer optimization and quenching protection. ACS Appl. Mater. Interface.

[41] Zhou J (2020). Single-molecule photoreaction quantitation through intraparticle-surface energy transfer (i-SET) spectroscopy. Nat. Commun..

[42] Wang Z (2014). Pure and intense orange upconversion luminescence of Eu^3+^ from the sensitization of Yb^3+^–Mn^2+^ dimer in NaY(Lu)F_4_ nanocrystals. J. Mater. Chem. C.

[43] Prorok K (2019). Near-infrared excited luminescence and in vitro imaging of HeLa cells by using Mn^2+^ enhanced Tb^3+^ and Yb^3+^ cooperative upconversion in NaYF_4_ nanocrystals. Nanoscale Adv..

[44] Chen X (2016). Confining energy migration in upconversion nanoparticles towards deep ultraviolet lasing. Nat. Commun..

[45] Bogdan N, Vetrone F, Ozin GA, Capobianco JA (2011). Synthesis of ligand-free colloidally stable water dispersible brightly luminescent lanthanide-doped upconverting nanoparticles. Nano Lett..

[46] Han S (2016). Multicolour synthesis in lanthanide-doped nanocrystals through cation exchange in water. Nat. Commun..

[47] Rabouw FT (2018). Quenching pathways in NaYF_4_:Er^3+^,Yb^3+^ upconversion nanocrystals. ACS Nano.

[48] Siefe C (2019). Sub-20 nm core–shell–shell nanoparticles for bright upconversion and enhanced Förster resonant energy transfer. J. Am. Chem. Soc..

[49] Marin R (2018). Upconverting nanoparticle to quantum dot Förster resonance energy transfer: increasing the efficiency through donor design. ACS Photon..

[50] Wang X (2017). Dye-sensitized lanthanide-doped upconversion nanoparticles. Chem. Soc. Rev..

[51] Su Q, Feng W, Yang D, Li F (2017). Resonance energy transfer in upconversion nanoplatforms for selective biodetection. Acc. Chem. Res..

[52] Chen G (2015). Energy-cascaded upconversion in an organic dye-sensitized core/shell fluoride nanocrystal. Nano Lett..

[53] Chen G (2016). Efficient broadband upconversion of near–infrared light in dye–sensitized core/shell nanocrystals. Adv. Optic. Mater..

[54] Wu Y, Xu J, Qin X, Xu J, Liu X (2021). Dynamic upconversion multicolour editing enabled by molecule-assisted opto-electrochemical modulation. Nat. Commun..

[55] Alyatkin S (2018). In-depth analysis of excitation dynamics in dye-sensitized upconversion core and core/active shell nanoparticles. J. Phys. Chem. C.

[56] Xue B (2018). Ultrastrong absorption meets ultraweak absorption: unraveling the energy-dissipative routes for dye-sensitized upconversion luminescence. J. Phys. Chem. Lett..

[57] Han S (2020). Lanthanide-doped inorganic nanoparticles turn molecular triplet excitons bright. Nature.

[58] Zhang Y, Huang L, Liu X (2016). Unraveling epitaxial habits in the NaLnF4 system for color multiplexing at the single-particle level. Angew. Chem. Int. Ed..

[59] Liu X (2017). Hedgehog-like upconversion crystals: controlled growth and molecular sensing at single-particle level. Adv. Mater..

[60] Huang X (2011). Encapsulating a ternary europium complex in a silica/polymer hybrid matrix for high performance luminescence application. J. Phys. Chem. C.

[61] Galaup C, Couchet J-M, Bedel S, Tisnès P, Picard C (2005). Direct access to terpyridine-containing polyazamacrocycles as photosensitizing ligands for Eu(III) luminescence in aqueous media. J. Org. Chem..

[62] Frisch, M. J. et al. *Gaussian 16, Revision A.03* (Gaussian, Inc, 2016).

[63] Zhao Y, Truhlar DG (2008). The M06 suite of density functionals for main group thermochemistry, thermochemical kinetics, noncovalent interactions, excited states, and transition elements: two new functionals and systematic testing of four M06-class functionals and 12 other functionals. Theor. Chem. Acc..

[64] Hehre, W. J., Random, L., Schleyer, P. v. R. & Pople, J. A. *Ab Initio Molecular Orbital Theory* (Wiley, 1986).

