# Supplementary material for: Photon upconversion through triplet exciton-mediated energy relay
Source: Nat Commun. 2021 Jun 17;12:3704. doi: 10.1038/s41467-021-23967-3 (PMC8211736; doi:10.1038/s41467-021-23967-3)
Supplement: Supplementary file 1 — Supplementary Information [file 41467_2021_23967_MOESM1_ESM.pdf]

Supplementary Information

Photon upconversion through triplet exciton-mediated energy relay

Sanyang Han[1,†], Zhigao Yi[1,†], Jiangbin Zhang[2,3], Qifei Gu[2], Liangliang Liang[1], Xian Qin[1]*, Jiahui Xu[1], Yiming Wu[1], Hui Xu[4]*, Akshay Rao[2]* and Xiaogang Liu[1,5,6]*

[1]*Department of Chemistry, National University of Singapore, Singapore 117543, Singapore.*

[2]*Department of Physics, Cavendish Laboratory, University of Cambridge, 19 JJ Thompson Avenue, Cambridge, CB3 0HE, United Kingdom.*

[3]*College of Advanced Interdisciplinary Studies, National University of Defense Technology, Changsha, 410073, China.*

[4]*Key Laboratory of Functional Inorganic Material Chemistry, Ministry of Education, School of Chemistry and Material Science, Heilongjiang University, 74 Xuefu Road, Harbin 150080, China.*

[5]*Joint School of National University of Singapore and Tianjin University, International Campus of Tianjin University, Fuzhou 350207, China.*

[6]*Center for Functional Materials, National University of Singapore Suzhou Research Institute, Suzhou 215123, China.*

[†]*These authors contributed equally.*

*Corresponding author. E-mail: chmqinx@nus.edu.sg; hxu@hlju.edu.cn; ar525@cam.ac.uk; chmlx@nus.edu.sg

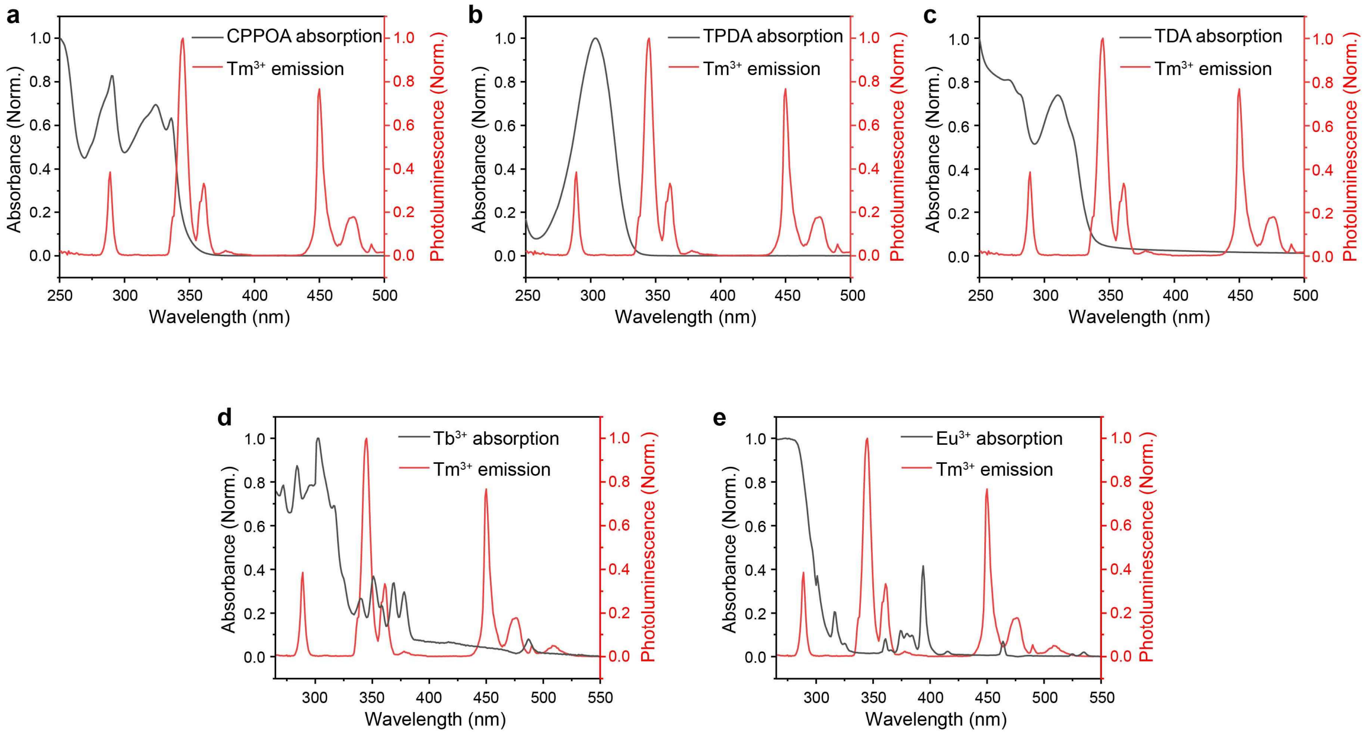

Supplementary Figure 1. **Spectroscopic investigations of donor emission and acceptor absorption.** Normalized Tm^{3+} emission spectra and absorption spectra of (**a**) CPPOA, (**b**) TPDA, (**c**) TDA, (**d**) Tb^{3+} and (**e**) Eu^{3+} acceptor.

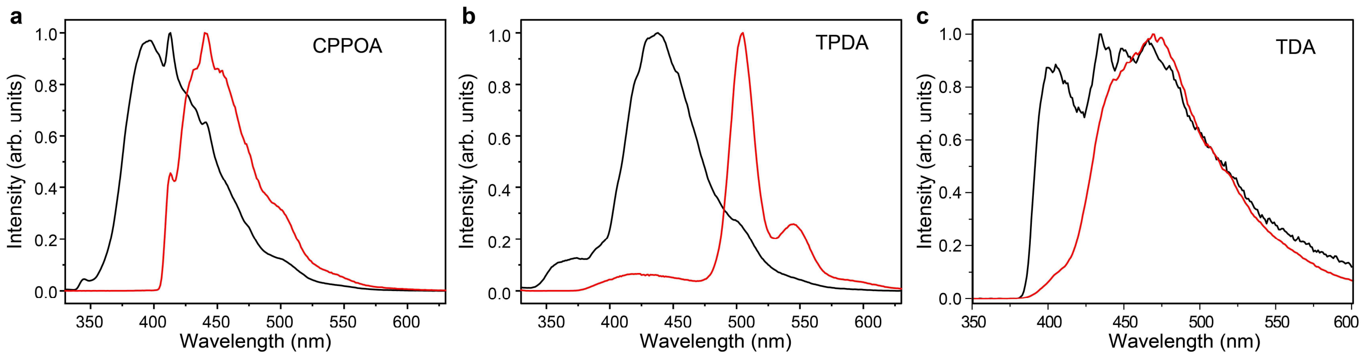

Supplementary Figure 2. **Photoluminescence spectra of the molecules under investigation**. Steady-state fluorescence (black) and phosphorescence (red) spectra of (**a**) CPPOA, (**b**) TPDA and (**c**) TDA molecules under 320-nm excitation. Phosphorescence spectra were recorded 8 ms after cessation of the excitation at 77 K.

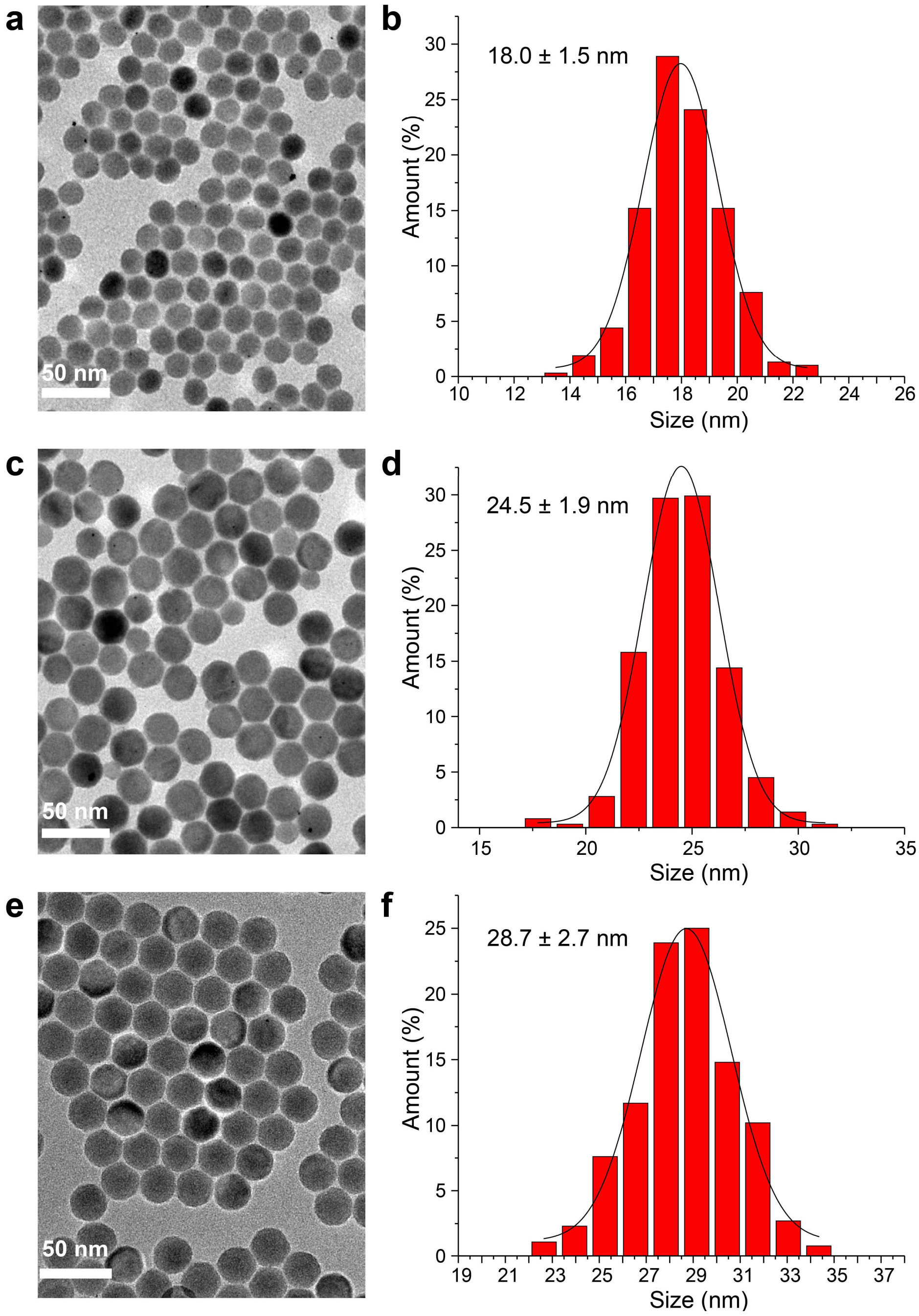

Supplementary Figure 3. Structural characterization of nanoparticles. Low-resolution TEM images and corresponding size histograms of (**a, b**) $NaYF_4$ core, (**c, d**) $NaYF_4$@$NaYbF_4$:1%Tm core-shell, and (**e, f**) $NaYF_4$@$NaYbF_4$:1%Tm@$NaYF_4$ core-shell-shell nanocrystals.

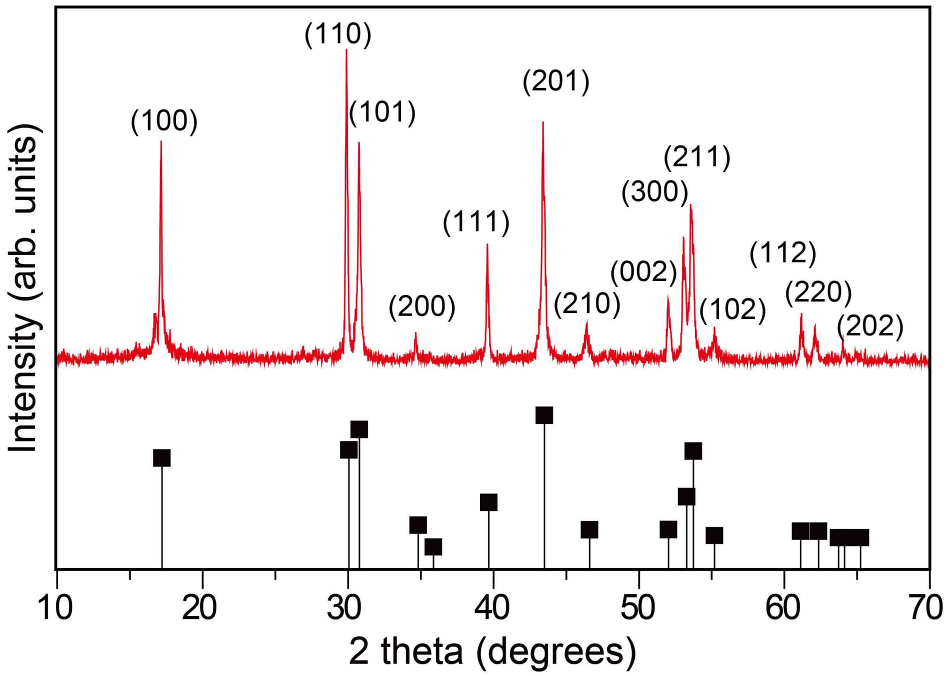

Supplementary Figure 4. X-ray powder diffraction (XRD) patterns of as-prepared $NaYF_4$@$NaYbF_4$:Tm@$NaYF_4$ nanoparticles.

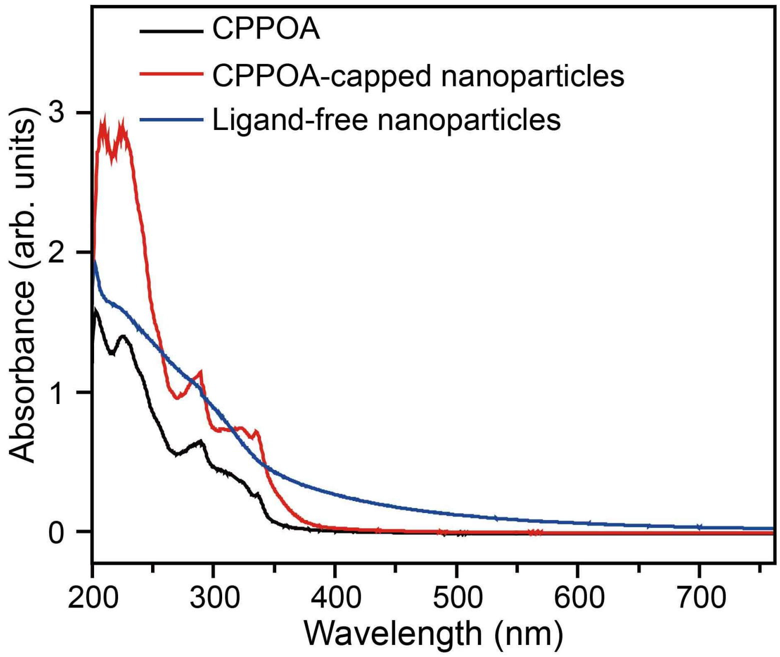

Supplementary Figure 5. UV-Vis absorption spectra of CPPOA, ligand-free $NaYF_4$@$NaYbF_4$:Tm@$NaYF_4$ and CPPOA-capped $NaYF_4$@$NaYbF_4$:Tm@$NaYF_4$ nanoparticles dispersed in ethanol.

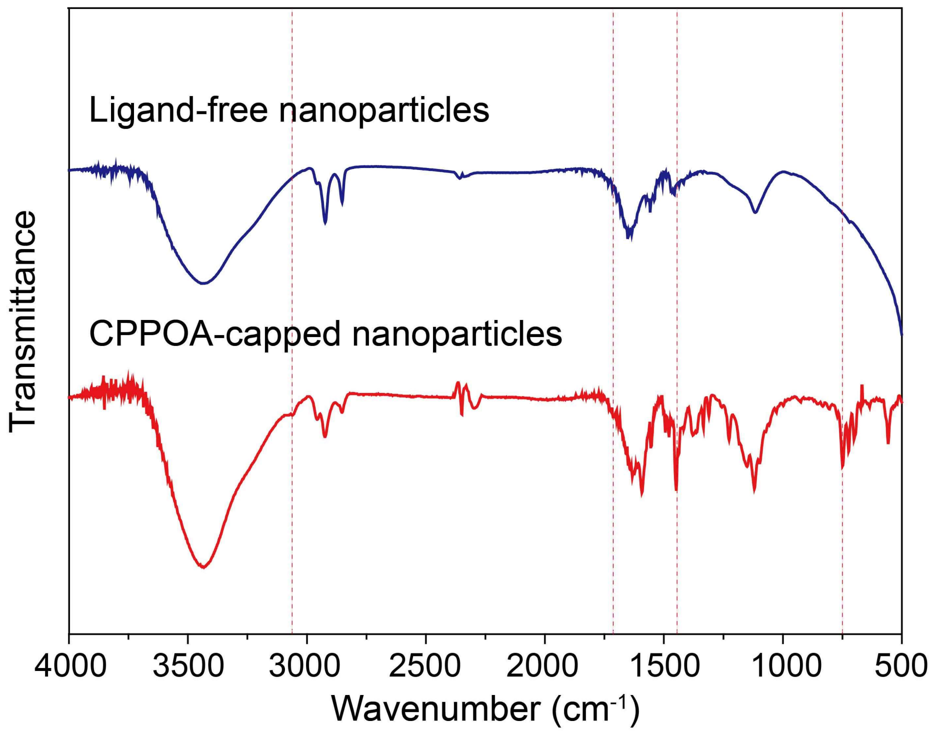

Supplementary Figure 6. Fourier-transform infrared spectra of CPPOA, ligand-free $NaYF_4$@$NaYbF_4$:Tm@$NaYF_4$ nanoparticles and CPPOA-capped $NaYF_4$@$NaYbF_4$:Tm@$NaYF_4$ nanoparticles.

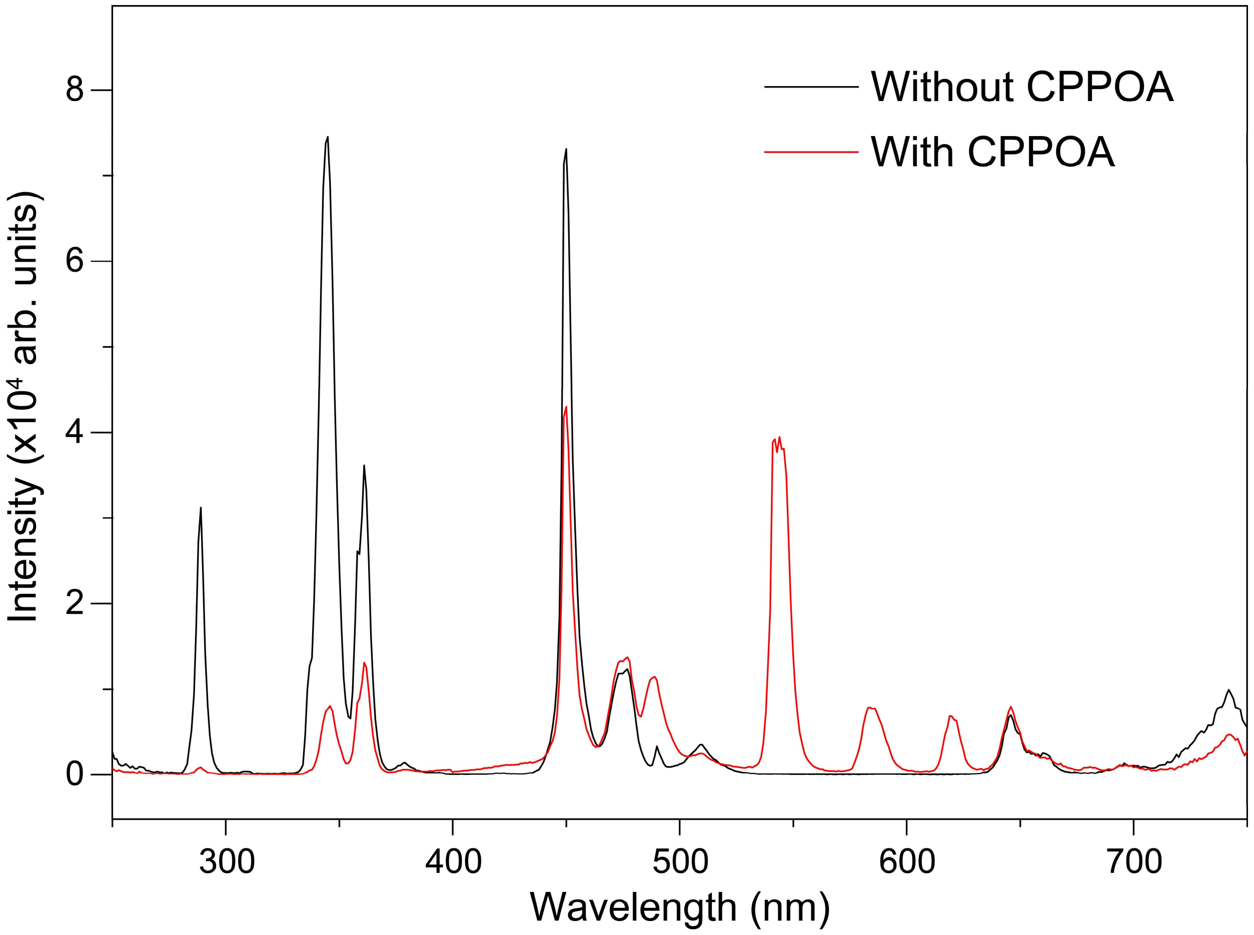

Supplementary Figure 7. Upconversion luminescence spectra of $NaYF_4$@$NaYbF_4$:1%Tm@$NaYF_4$:Tb multilayer nanocrystals with and without CPPOA modification (excitation, 980 nm).

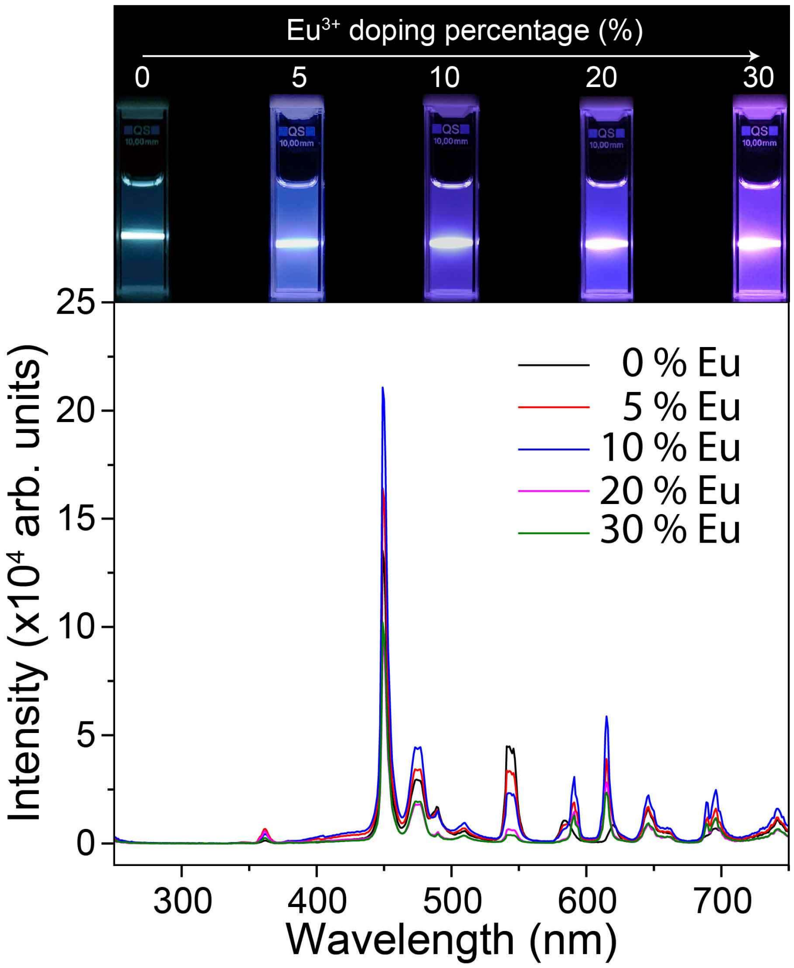

Supplementary Figure 8. Effect of Eu^{3+} doping concentration on upconversion luminescence. Upconversion emission spectra of $TbCl_3$-treated $NaYF_4$@$NaYbF_4$:Tm (1 mol%)@$NaYF_4$:Eu nanoparticles with CPPOA modification plotted versus Eu^{3+} doping concentration (excitation, 980 nm). Insets are corresponding luminescence photographs.

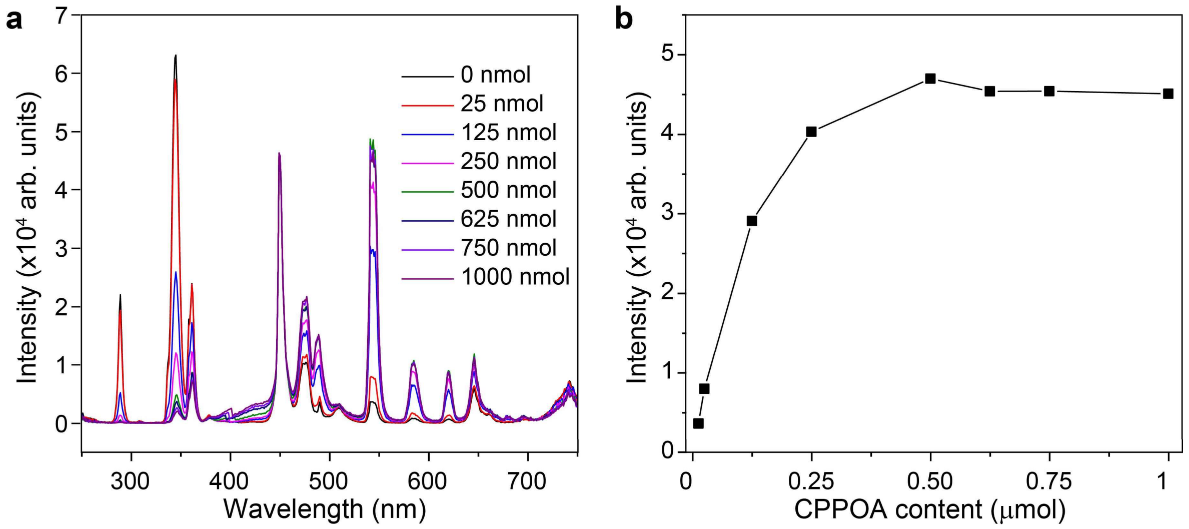

Supplementary Figure 9. Effect of ligand content on energy relay upconversion luminescence. a, Upconversion emission spectra of CPPOA-modified $NaYF_4$@$NaYbF_4$:Tm@$NaYF_4$:Tb nanoparticles plotted versus the amount of CPPOA (excitation, 980 nm). **b,** Intensity change in Tb^{3+} emission at 546 nm as a function of CPPOA content.

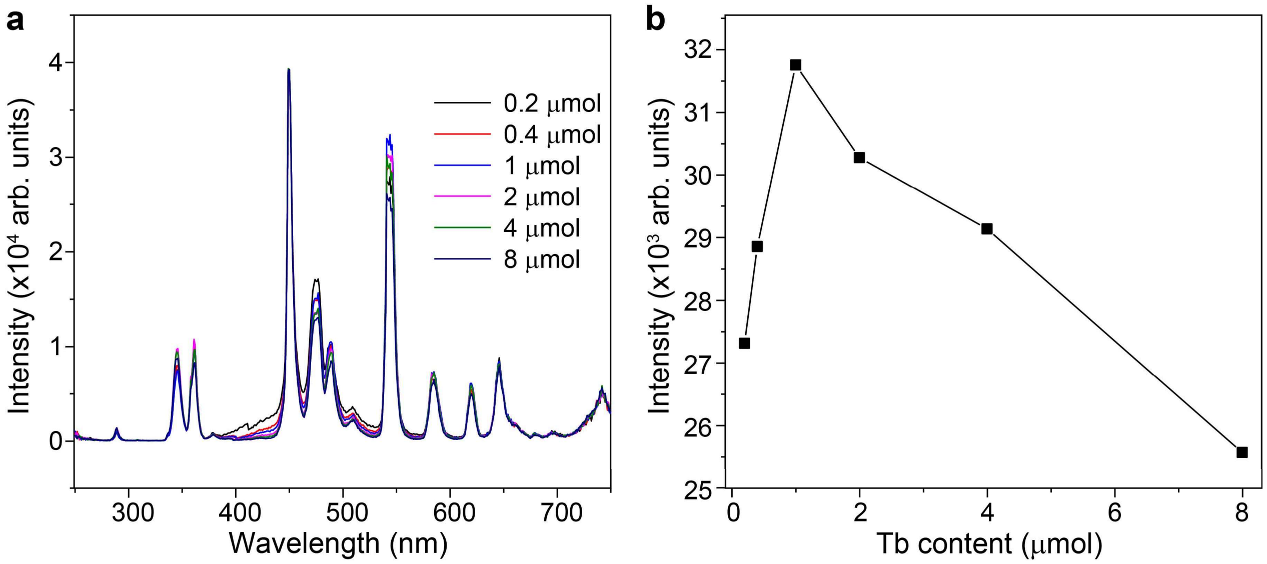

Supplementary Figure 10. Effect of activator content on energy relay upconversion luminescence. a, Upconversion emission spectra of CPPOA-modified $NaYF_4$@$NaYbF_4$:Tm@$NaYF_4$ nanoparticles plotted versus Tb^{3+} concentration used for cation exchange (excitation, 980 nm). **b,** Tb^{3+} emission at 546 nm changes as a function of Tb^{3+} content.

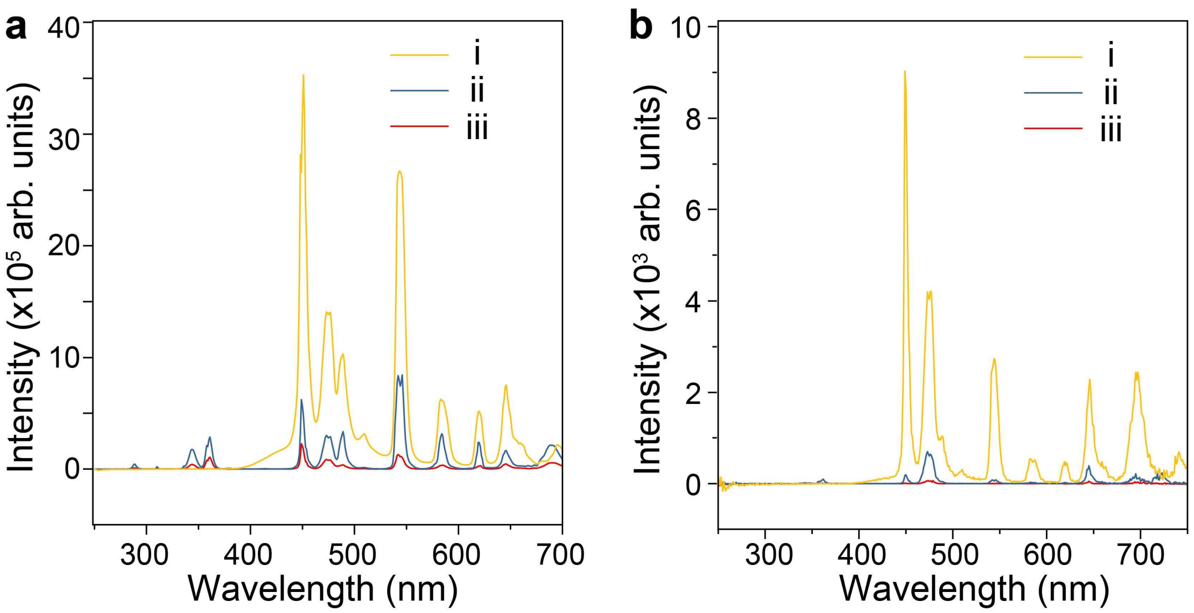

Supplementary Figure 11. Upconversion emission through triplet exciton-mediated energy relay. Upconversion luminescence spectra of CPPOA-modified (i) $NaYF_4$@$NaYbF_4$:1%Tm@$NaYF_4$:Tb, (ii) $NaGdF_4$:Yb/Tm(49/1 mol%)@$NaGdF_4$:Tb(15 mol%), and (iii) $NaGdF_4$:Yb/Tm(49/1 mol%)@$NaGdF_4$ (cation exchange with 5-µmol $TbCl_3$) nanocrystals (excitation, 980 nm; power density, 507 and 76 W/cm^2 for **a** and **b**, respectively).

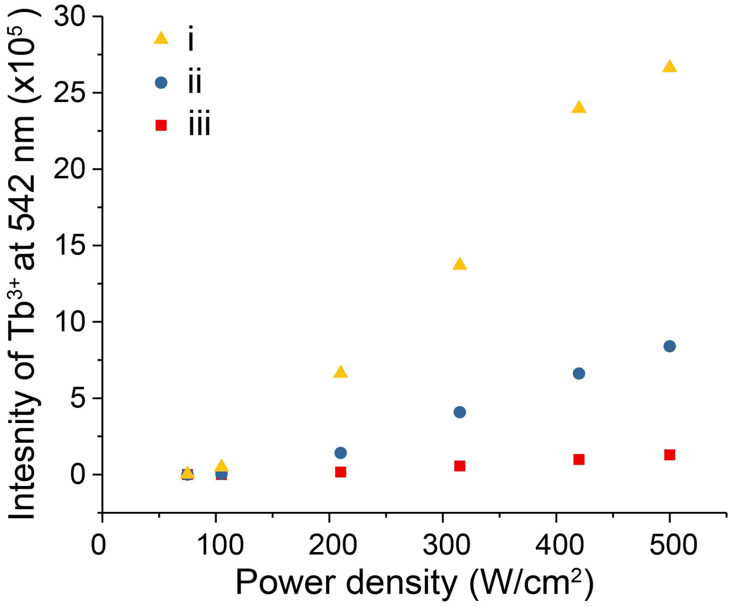

Supplementary Figure 12. Power-dependent upconversion luminescence. Intensity change in Tb^{3+} emission (542 nm) versus the power density of CPPOA-modified (i) $NaYF_4$@$NaYbF_4$:1%Tm@$NaYF_4$:Tb, (ii) $NaGdF_4$:Yb/Tm(49/1 mol%)@$NaGdF_4$:Tb(15 mol%), and (iii) $NaGdF_4$:Yb/Tm(49/1 mol%)@$NaGdF_4$ (cation exchange with 5-µmol $TbCl_3$) nanocrystals.

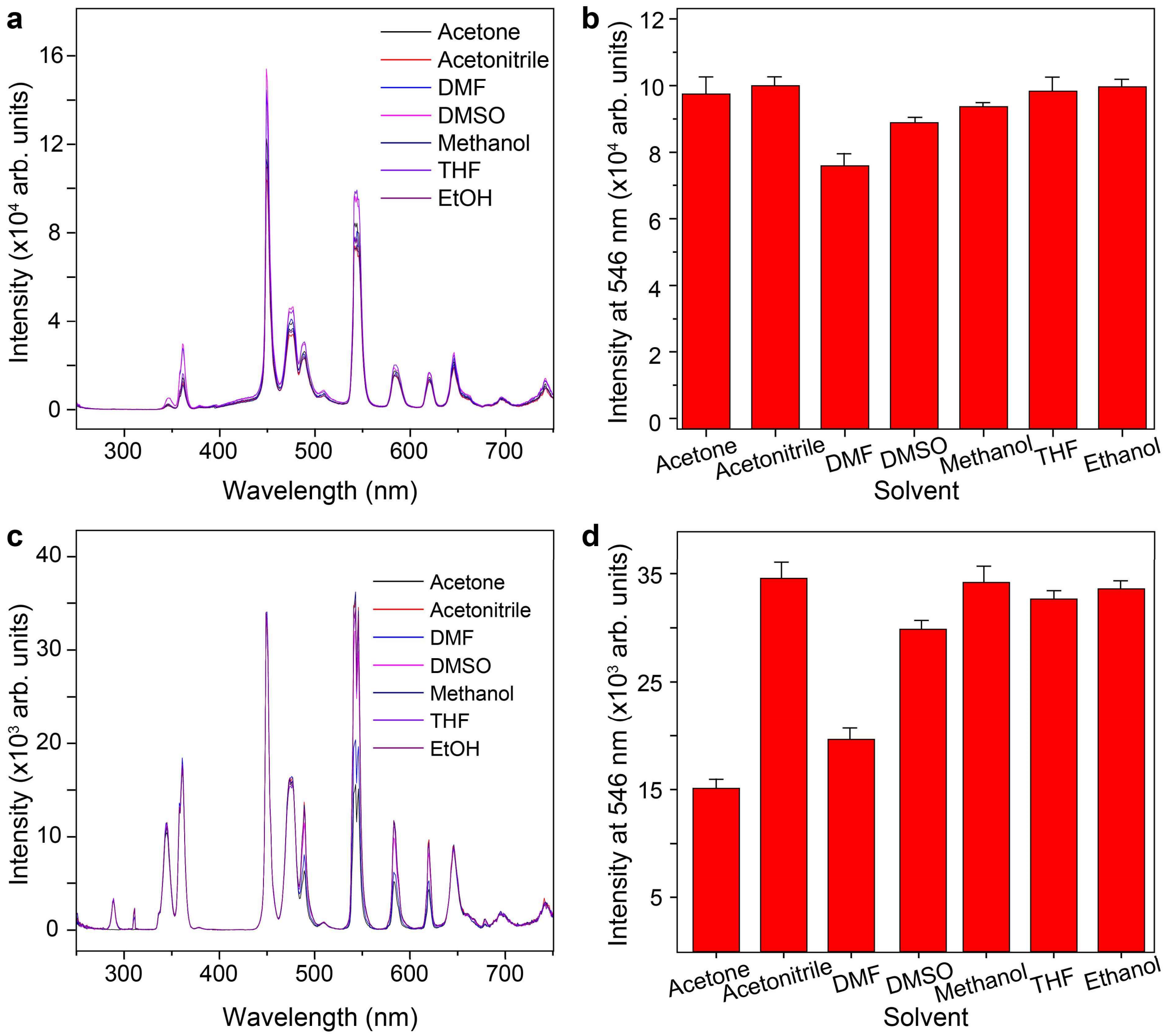

Supplementary Figure 13. Effect of solvent on energy relay upconversion luminescence. a, Upconversion luminescence spectra of CPPOA-modified $NaYF_4$@$NaYbF_4$:1%Tm@$NaYF_4$:Tb nanocrystals dispersed in a variety of solvents (excitation, 980 nm). **b**, Corresponding intensity histograms of Tb^{3+} emission at 546 nm. **c**, Upconversion luminescence spectra of ligand-free $NaGdF_4$:Yb/Tm(49/1 mol%)@$NaGdF_4$:Tb(10 mol%) nanocrystals dispersed in different solvents (excitation, 980 nm). **d**, Corresponding intensity histograms of Tb^{3+} emission at 546 nm.

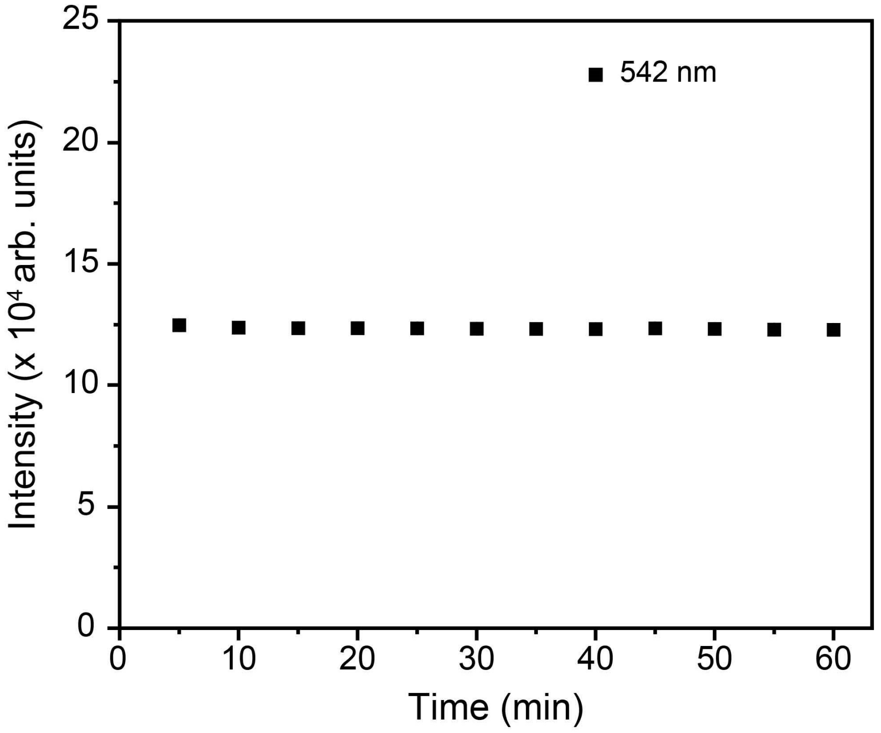

Supplementary Figure 14. Photostability investigation. Upconversion emission intensity of Tb^{3+} at 542 nm from CPPOA-modified $NaYF_4$@$NaYbF_4$:Tm(1%)@$NaYF_4$ nanoparticles in ethanol under 980-nm irradiation (500 W/cm^2).

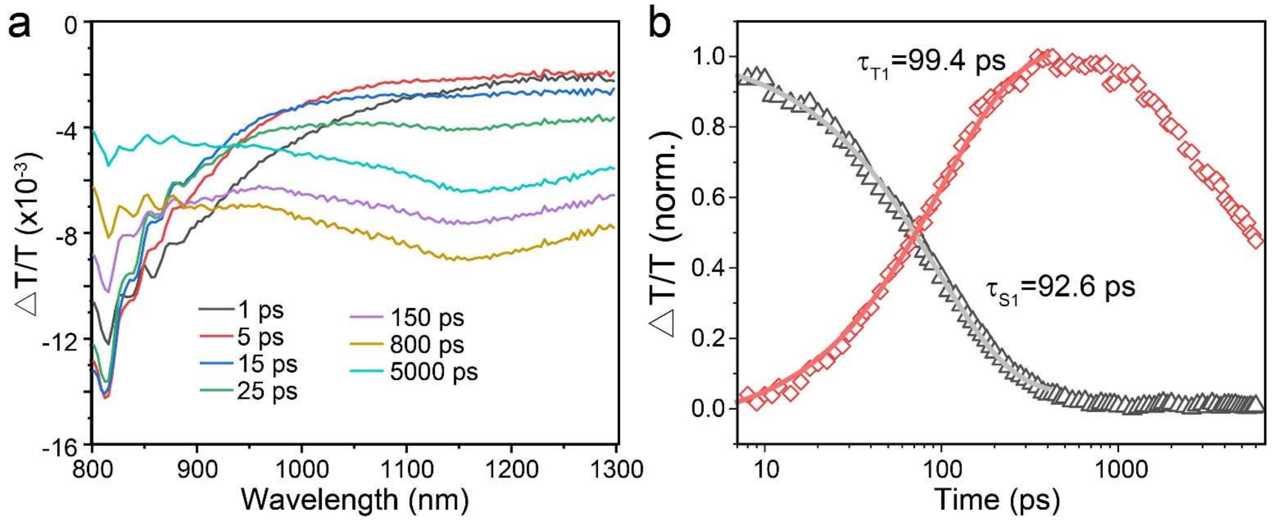

Supplementary Figure 15. Femtosecond transient absorption spectroscopy studies. **a,** Transient absorption spectra of CPPOA-modified $NaYF_4$@$NaYbF_4$:1%Tm@$NaYF_4$:Tb nanoparticles obtained by cation exchange. The excitation wavelength is 290 nm with a pulse energy of 2 μJ. **b**, Kinetics of singlet (S_1) decay and triplet (T_1) rise, obtained from picosecond transient absorption of CPPOA-modified colloidal solutions.

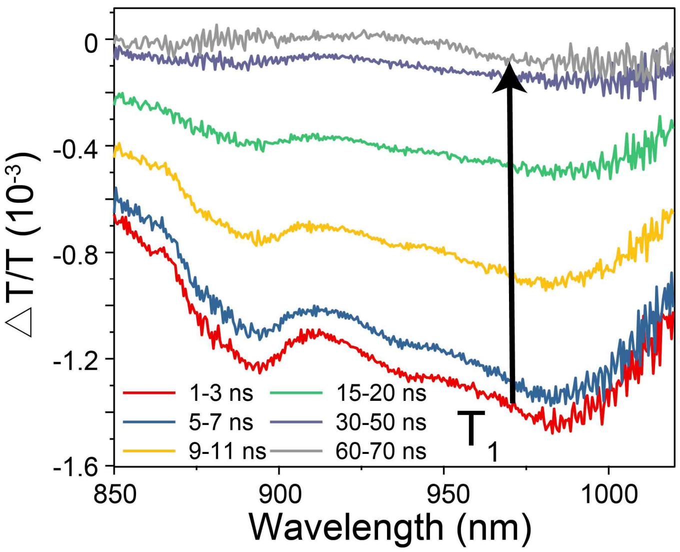

Supplementary Figure 16. Nanosecond transient absorption spectra of CPPOA-modified $NaYF_4$@$NaYbF_4$:Tm@$NaYF_4$:Tb nanocrystals probed in near-infrared spectral range (850-1020 nm). The pump wavelength is 355 nm.

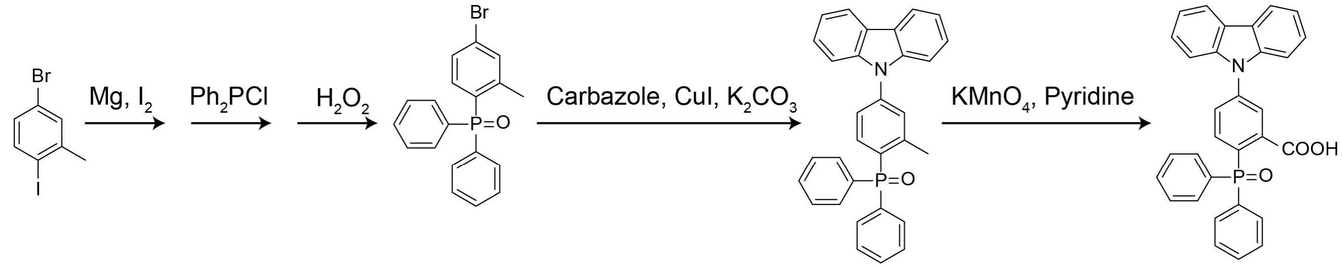

TPPOMBr CPPOM CPPOA

Supplementary Figure 17. The synthetic scheme of CPPOA molecule.

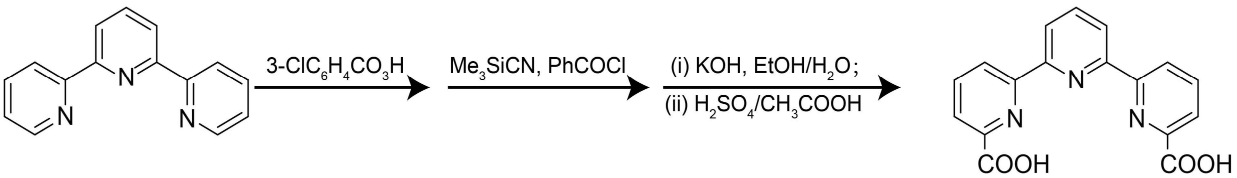

TDA

Supplementary Figure 18. The synthetic scheme of TDA molecule.

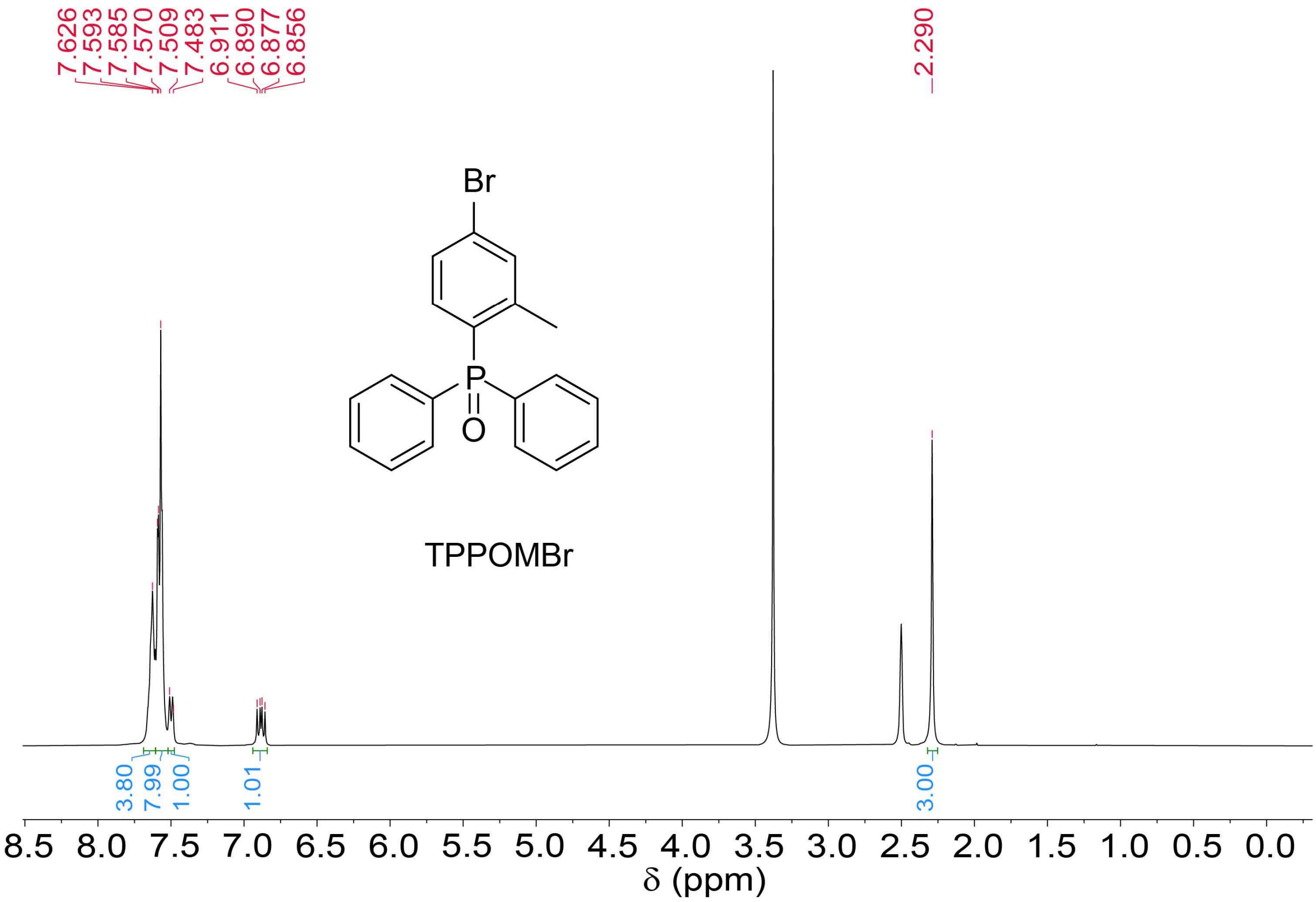

Supplementary Figure 19. ^{1}H NMR spectrum of TPPOMBr molecule. ^{1}H NMR (TMS, DMSO-*d6*, 400 MHz): δ = 7.66-7.63 (m, 3H), 7.601-7.56 (m, 8H), 7.508 (d, J= 8.4 Hz, 1H), 6.91 (q, J_1=8.4 Hz, J_2=13.2 Hz, 1H), 2.29 ppm (s, 3H).

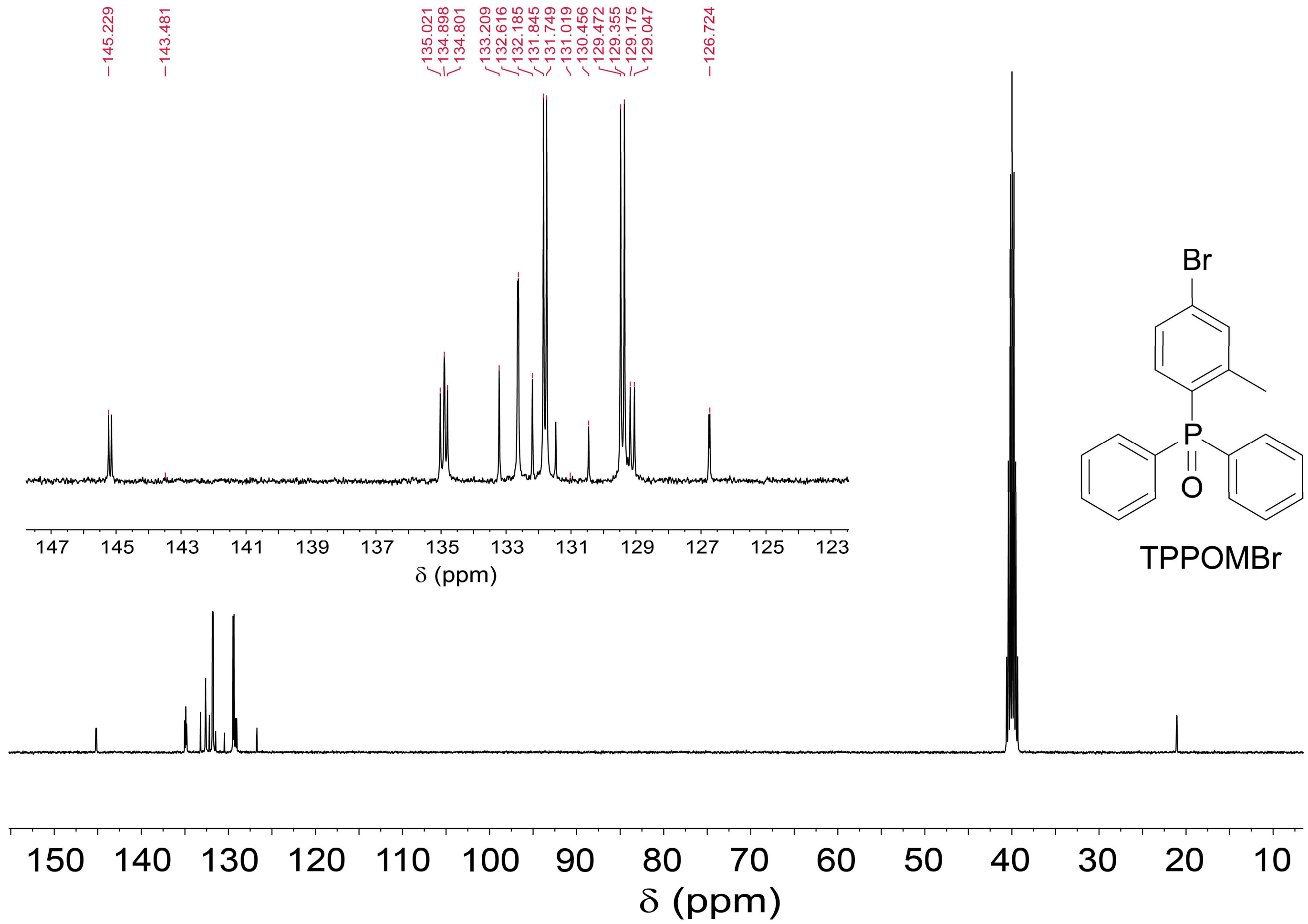

Supplementary Figure 20. ^{13}C NMR spectrum of TPPOMBr molecule. ^{13}C NMR (TMS, DMSO-*d6*, 100 MHz): δ = 145.2, 145.1, 135.0, 134.9, 134.8, 133.2, 132.7, 132.6, 132.2, 131.9, 131.8, 131.5, 130.5, 129.5, 129.4, 129.2, 129.1, 126.8, 126.7.

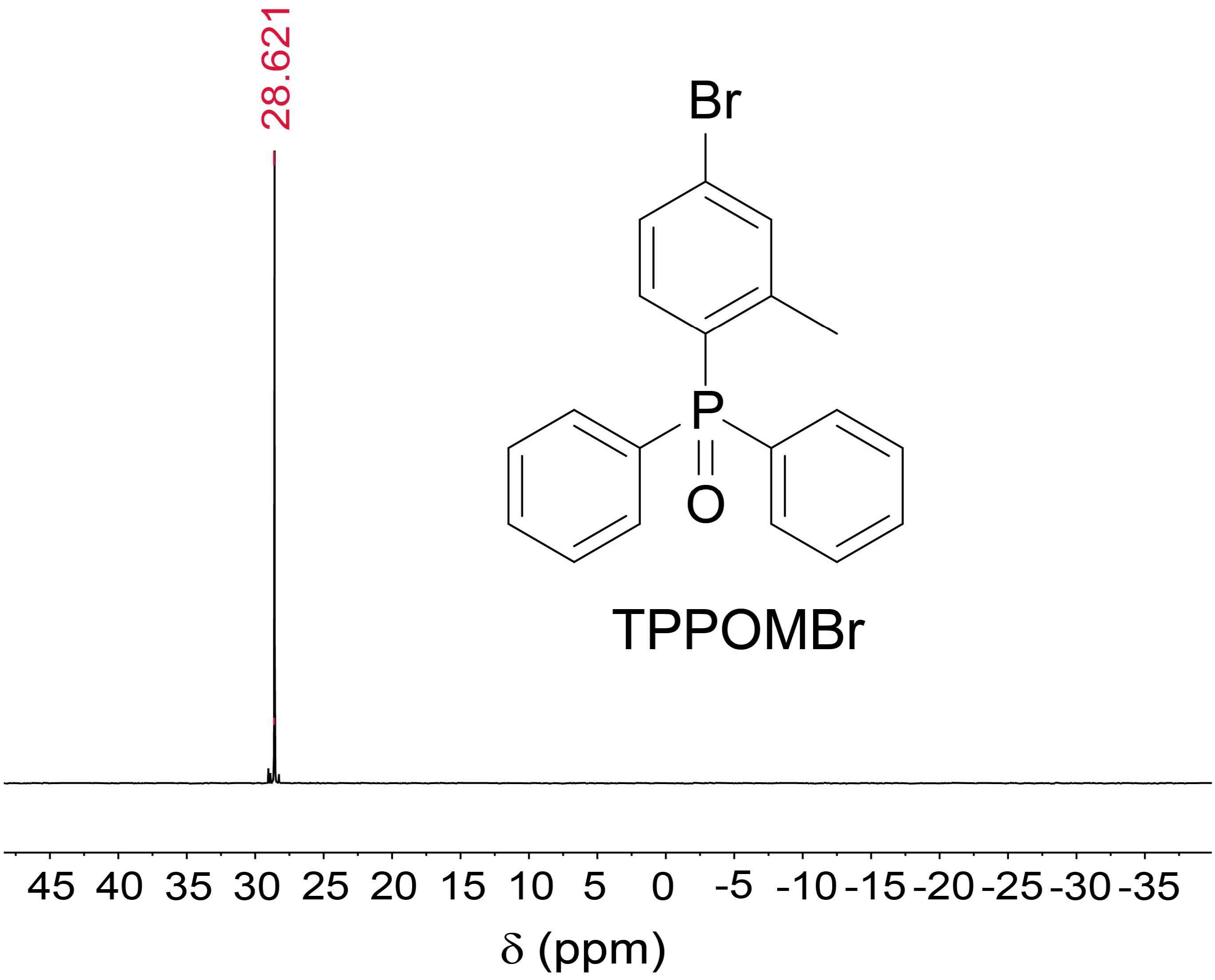

Supplementary Figure 21. ^{31}P NMR spectrum of TPPOMBr molecule. ^{31}P NMR (TMS, DMSO-*d6*, 121.5 MHz): δ = 28.6

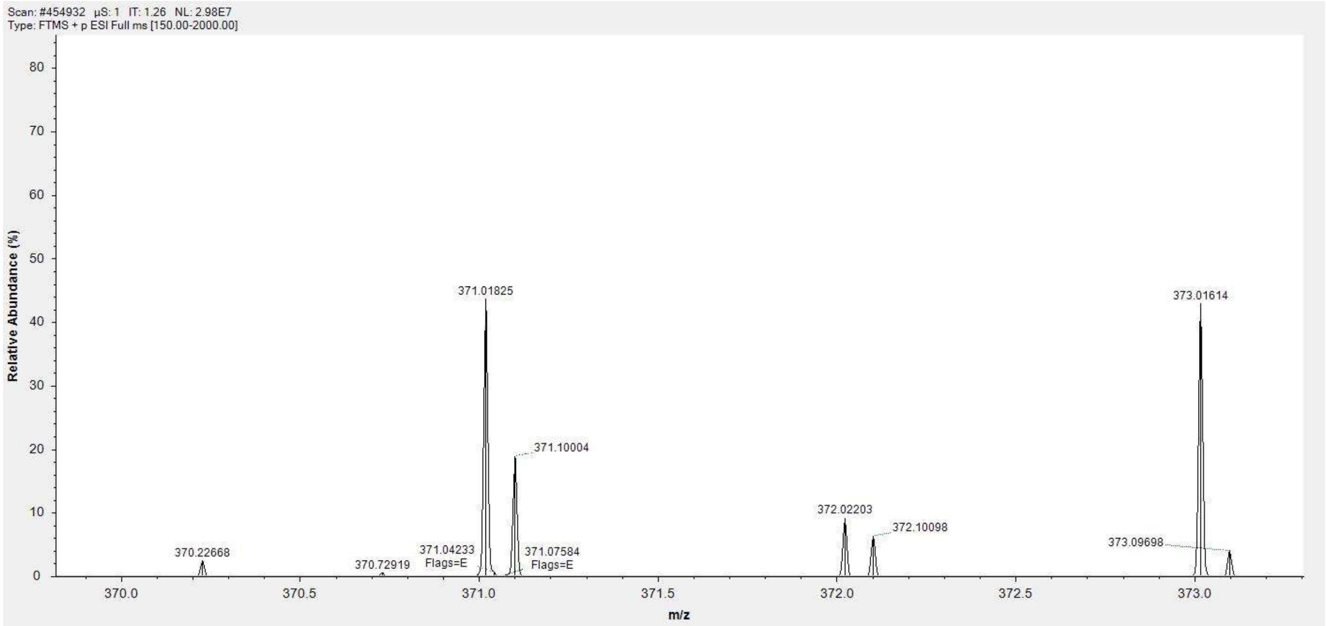

Supplementary Figure 22. High-resolution mass spectrum of TPPOMBr molecule.

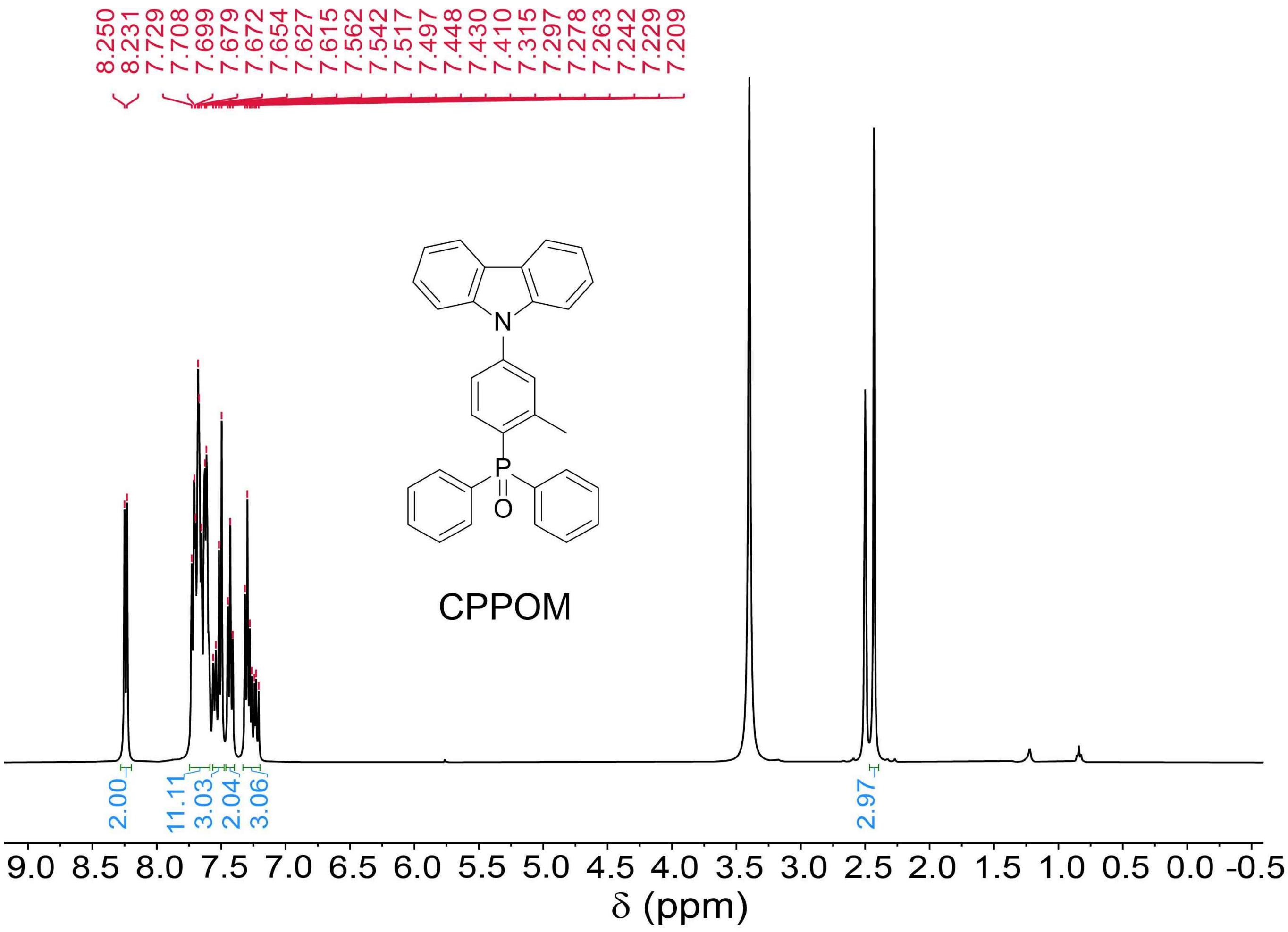

Supplementary Figure 23. ^{1}H NMR spectrum of CPPOM molecule. ^{1}H NMR (TMS, DMSO-d_6, 400 MHz): δ = 8.25 (d, J = 8.0 Hz, 2H), 7.73-7.60 (m, 11H), 7.56 (q, J_1 = 8.4 Hz, J_2 = 18 Hz, 3H), 7.45 (t, J = 7.6 Hz, 2H), 7.32-7.21 (m, 3H), 2.43 ppm (s, 3H)

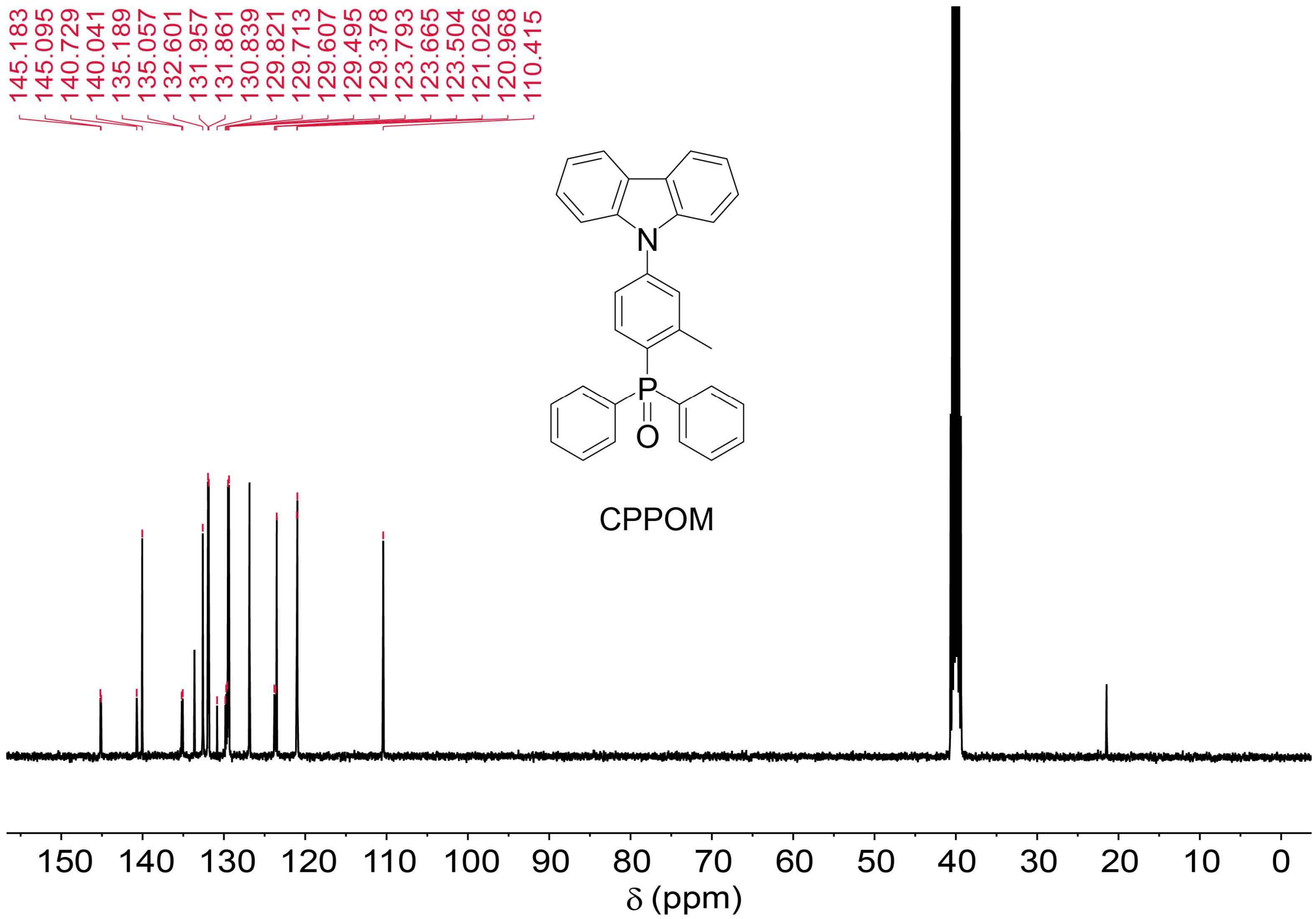

Supplementary Figure 24. ^{13}C NMR spectrum of CPPOM molecule. ^{13}C NMR (TMS, DMSO-d_6, 100 MHz): δ = 145.2, 145.1, 140.7, 140.7, 140.1, 135.2, 135.1, 133.6, 132.6, 132.0, 131.9, 130.9, 129.8, 129.8, 129.7, 129.6, 129.5, 129.4, 126.9, 123.8, 123.7, 123.5, 121.0, 120.9, 110.4.

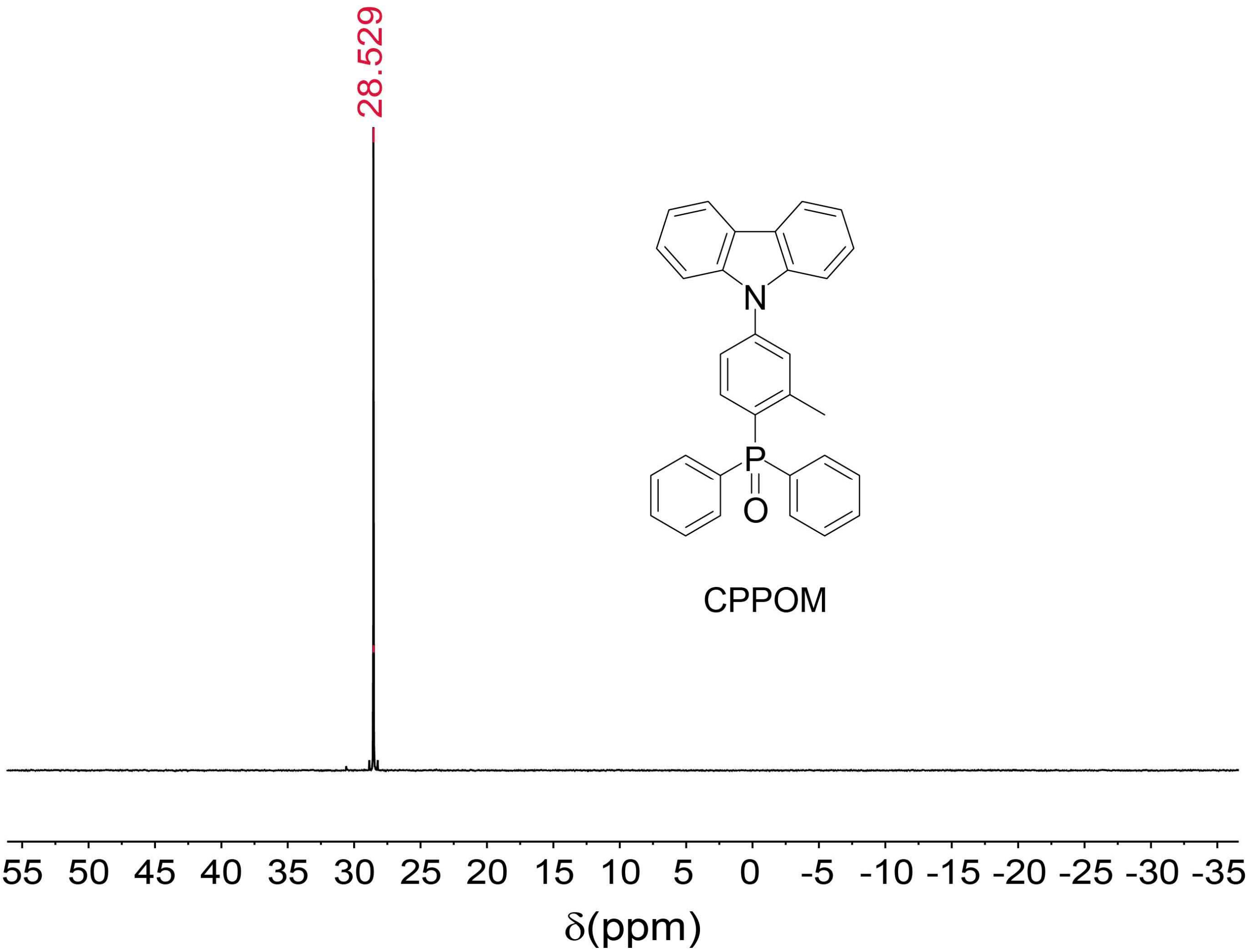

Supplementary Figure 25. ^{31}P NMR spectrum of CPPOM molecule. ^{31}P NMR (TMS, DMSO-d_6, 121.5 MHz): δ = 28.5

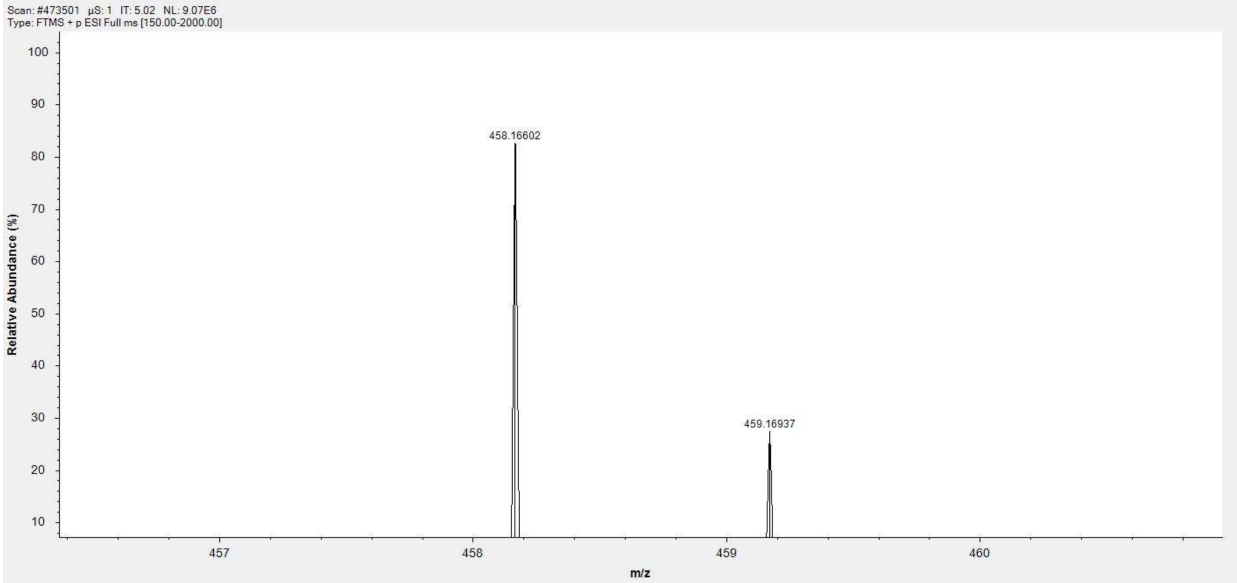

Supplementary Figure 26. High-resolution mass spectrum of CPPOM molecule.

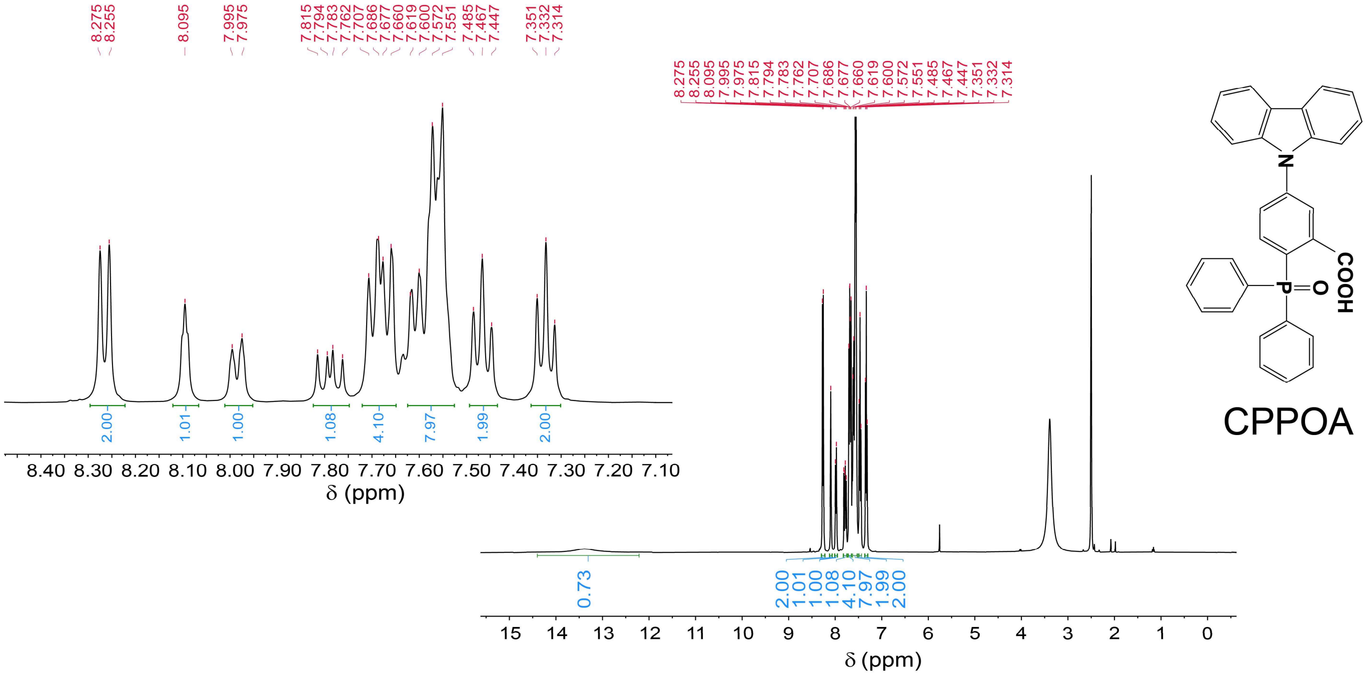

Supplementary Figure 27. ^{1}H NMR spectrum of CPPOA molecule. ^{1}H NMR (TMS, DMSO-d$_6$, 400 MHz): δ = 13.40 (s, 1H), 8.27 (d, J = 7.6Hz, 2H), 8.10 (t, J = 2.4 Hz, 1H), 7.99 (d, J = 8.4 Hz, 1H), 7.814 (q, J_1 = 8.4Hz, J_2 = 13.2 Hz, 1H), 7.71-7.66 (m, 4H), 7.63-7.65 (m, 8H), 7.484 (t, J = 7.6 Hz, 2H), 7.40 ppm (t, J = 7.2 Hz, 2H).

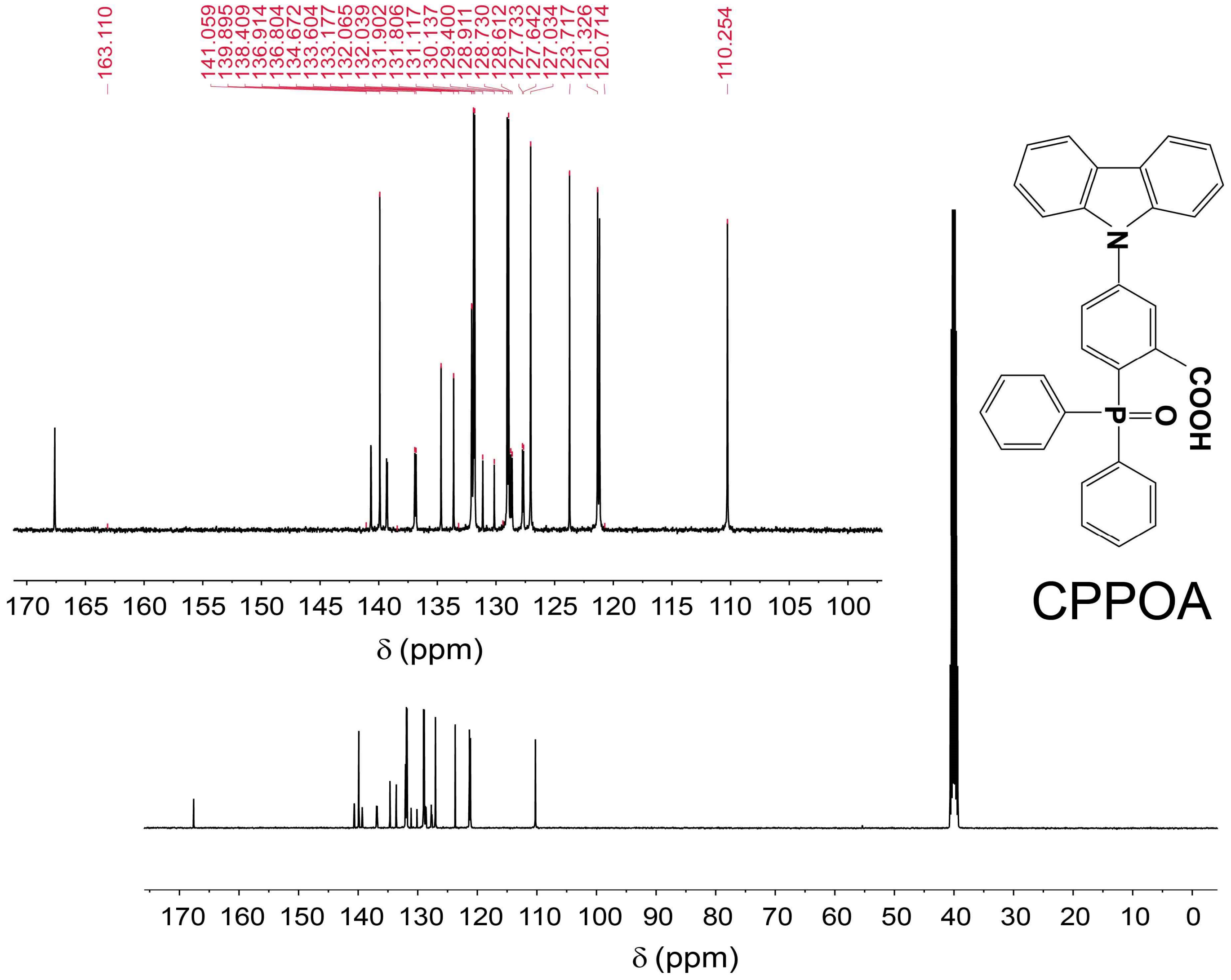

Supplementary Figure 28. ^{13}C NMR spectrum of CPPOA molecule. ^{13}C NMR (TMS, DMSO-d$_6$, 100 MHz): δ = 167.6, 167.6, 140.7, 140.6, 139.9, 139.3, 139.3, 136.9, 136.8, 134.7, 133.6, 132.1, 132.0, 131.9, 131.8, 131.1, 130.1, 129.0, 128.9, 128.7, 128.6, 127.7, 127.6, 127.0, 123.7, 121.1, 121.1, 110.3.

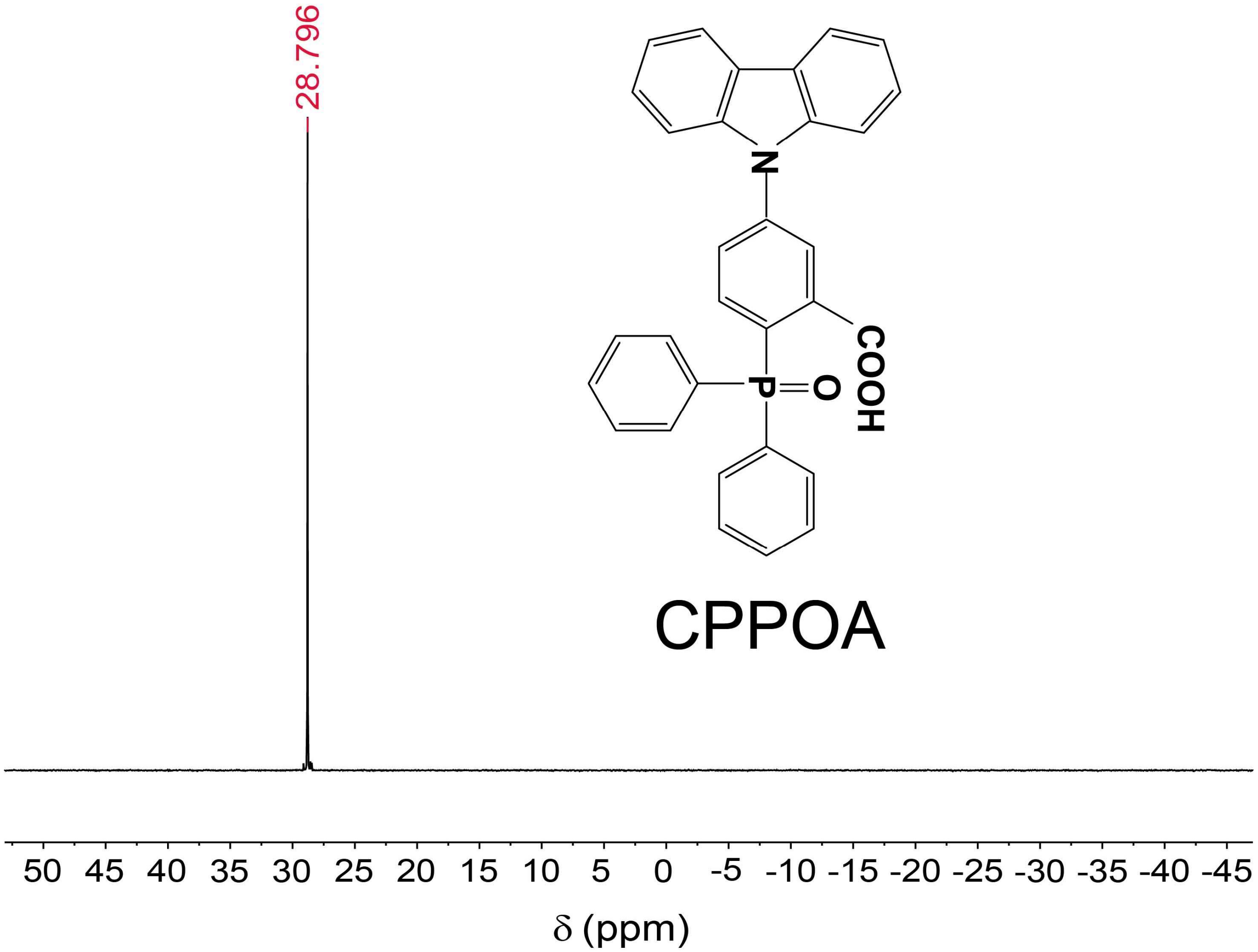

Supplementary Figure 29. ^{31}P NMR spectrum of CPPOA molecule. ^{31}P NMR (TMS, DMSO-d_6, 121.5 MHz): δ = 28.8

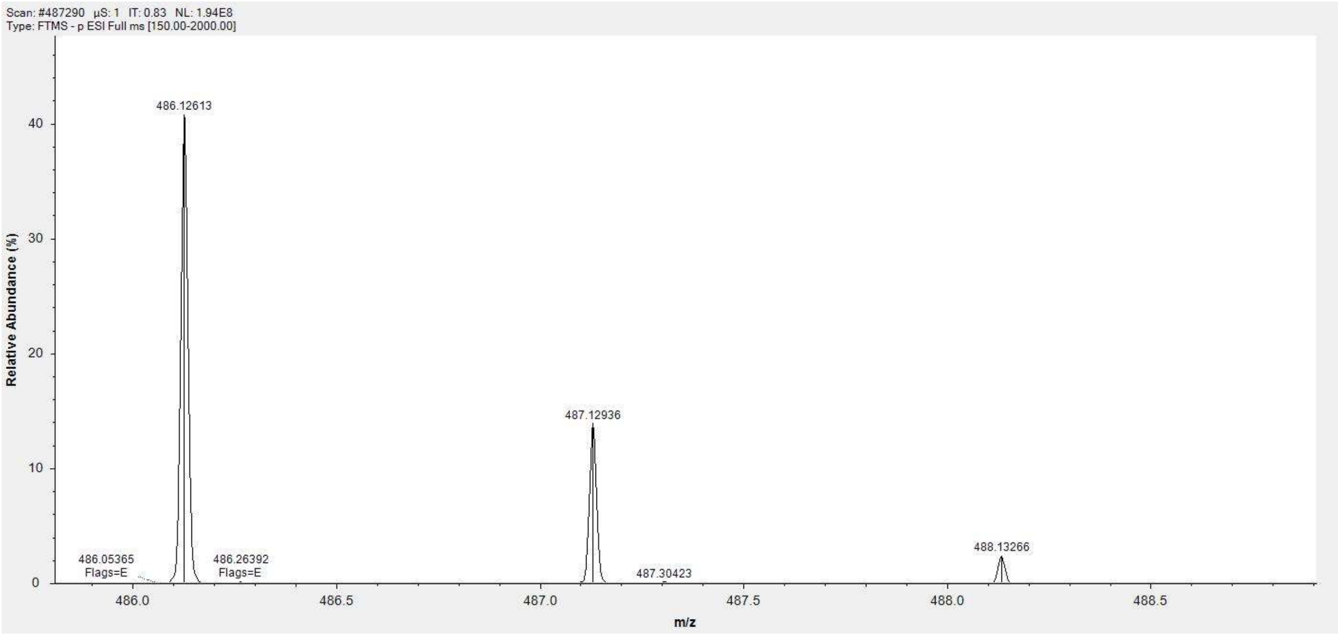

Supplementary Figure 30. High-resolution mass spectrum of CPPOA molecule.

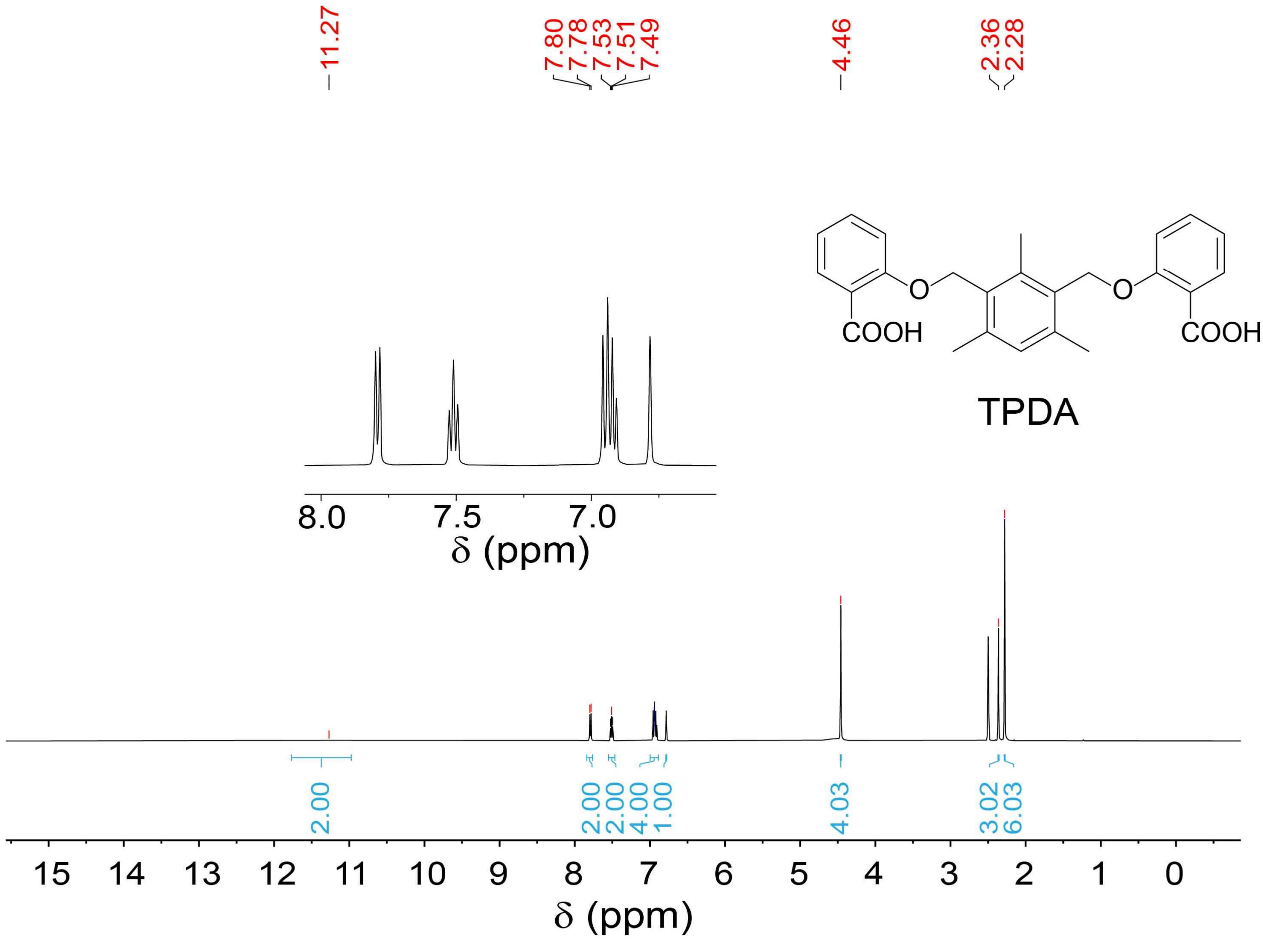

Supplementary Figure 31. ^{1}H NMR spectrum of TPDA molecule. ^{1}H NMR (500 MHz, DMSO-d_6) δ = 2.28 (s, 6H), 2.36 (s, 3H), 4.46 (s, 4H), 6.78 (s, 1H), 6.93 (dd, J = 9.0, 17.5 Hz, 4H), 7.51 (t, J = 7.5 Hz, 2H), 7.79 (d, J = 7.5 Hz, 2H), 11.27 (br, 2H).

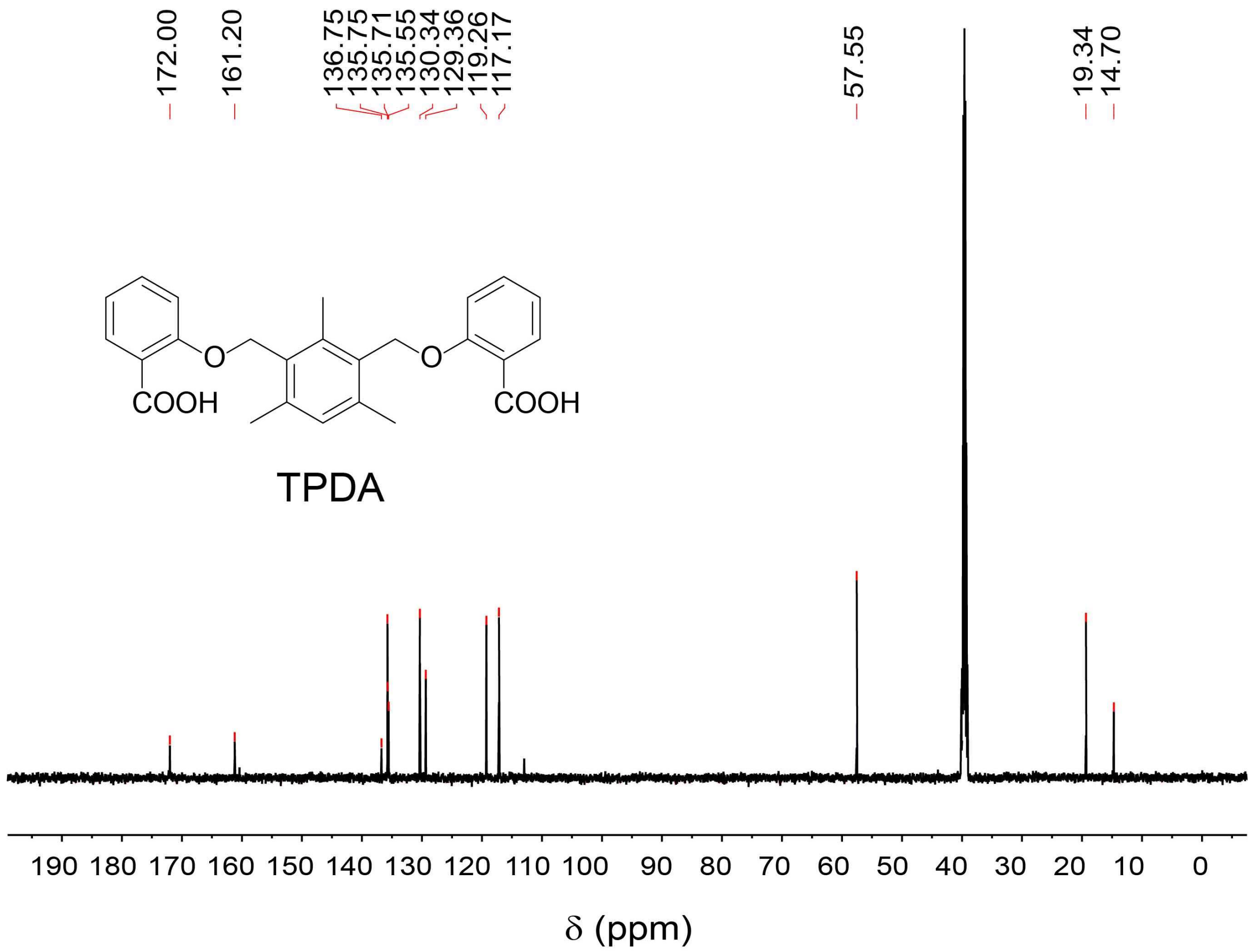

Supplementary Figure 32. ^{13}C NMR spectrum of TPDA molecule. ^{13}C NMR (125 MHz, DMSO-d_6) δ = 172.00, 161.20, 136.75, 135.75, 135.71, 135.55, 130.34, 129.36, 119.26, 117.17, 57.55, 19.34, 14.70.

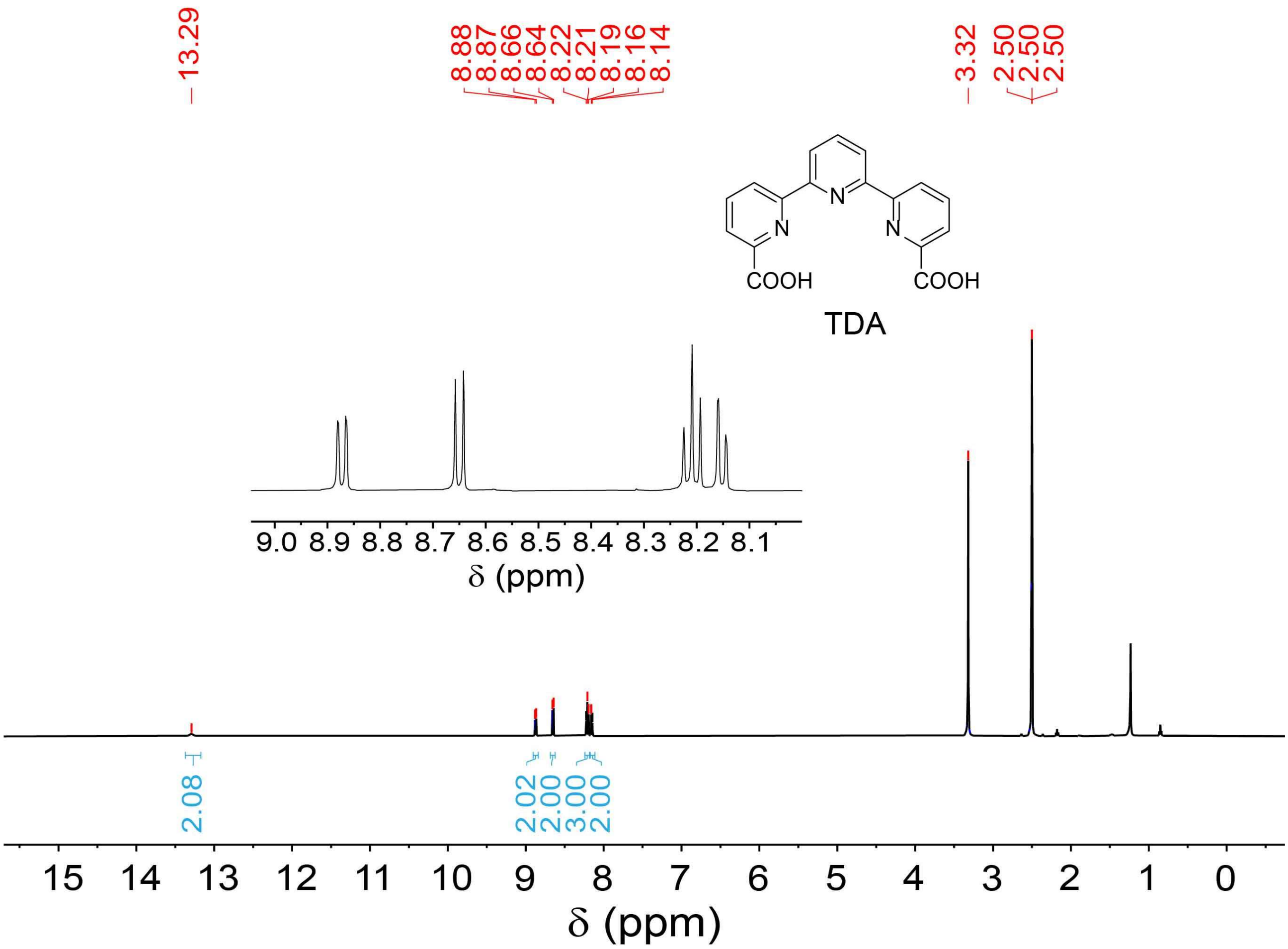

Supplementary Figure 33. ^{1}H NMR spectrum of TDA molecule. ^{1}H NMR (500 MHz, DMSO-d_6) δ = 8.18 (d, J = 6.5 Hz, 2H), 8.21 (t, J = 7.5 Hz, 3H), 8.65 (d, J = 7.5 Hz, 2H), 8.87 (d, J = 6.5 Hz, 2H), 13.29 (s, 2H).

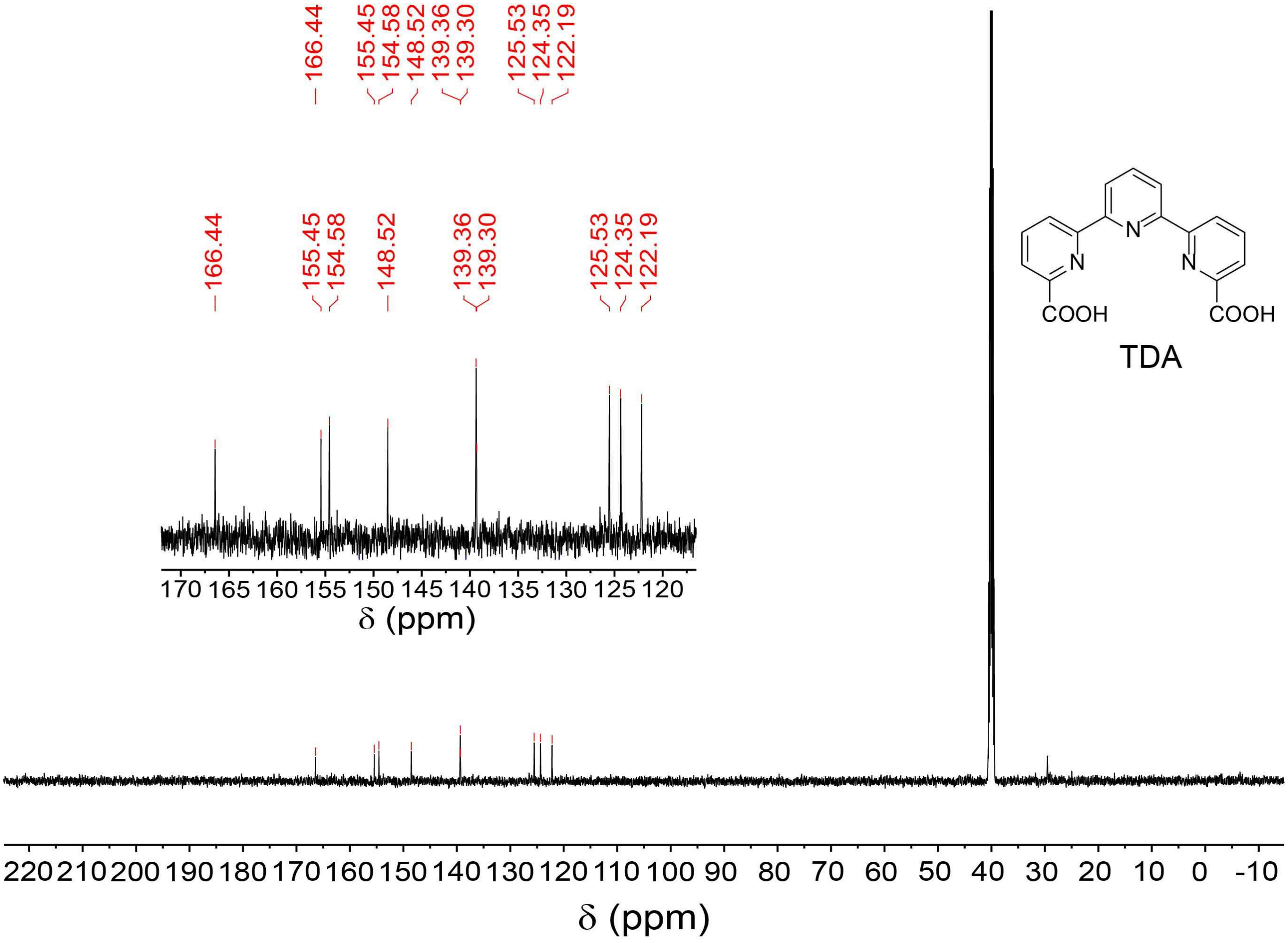

Supplementary Figure 34. ^{13}C NMR spectrum of TDA molecule. ^{13}C NMR (125 MHz, DMSO-d_6) δ = 166.44, 155.45, 154.58, 148.52, 139.36, 139.30, 125.53, 124.35, 122.19.

Supplementary Table 1. Calculated energy states of organic molecules.

	S_1	T_1
CPPOA	3.37 eV (368 nm)	2.53 eV (491 nm)
TPDA	3.83 eV (324 nm)	2.06 eV (600 nm)
TDA	3.10 eV (400 nm)	2.53 eV (490 nm)

Supplementary Table 2. Energy transitions and corresponding energy gaps of lanthanide emitters.

Lanthanides	Energy transitions			
Tm^{3+}	$^3H_6 \rightarrow {}^1D_2$<br>27830 cm^{-1}<br>(3.45 eV)	$^3H_6 \rightarrow {}^1I_2$<br>34684 cm^{-1}<br>(4.30 eV)		
Eu^{3+}	$^7F_1 \rightarrow {}^7D_2$<br>16936 cm^{-1}<br>(2.10 eV)	$^7F_2 \rightarrow {}^7D_2$<br>16268 cm^{-1}<br>(2.02 eV)	$^7F_4 \rightarrow {}^7D_2$<br>14420 cm^{-1}<br>(1.79 eV)	
Tb^{3+}	$^7F_6 \rightarrow {}^5D_4$<br>20471 cm^{-1}<br>(2.54 eV)	$^7F_5 \rightarrow {}^5D_4$<br>18433 cm^{-1}<br>(2.29 eV)	$^7F_4 \rightarrow {}^5D_4$<br>17175 cm^{-1}<br>(2.13 eV)	$^7F_3 \rightarrow {}^5D_4$<br>16201 cm^{-1}<br>(2.01 eV)

Supplementary Table 3. Förster distance of different donor-acceptor pairs.

Donor	Acceptor	Förster distance (R_o, nm)	Spectral overlap integral (J, $nm^4 \cdot M^{-1} \cdot cm^{-1}$)
Tm^{3+}	CPPOA	2.48	$2.29*10^{13}$
Tm^{3+}	TPDA	1.68	$2.21*10^{12}$
Tm^{3+}	TDA	2.2	$1.14*10^{13}$
Tm^{3+}	Tb^{3+}	0.58	$3.40*10^{9}$
Tm^{3+}	Eu^{3+}	0.64	$6.31*10^{9}$

Supplementary Table 4. Inductively coupled plasma optical emission spectroscopy (ICP-OES) analysis.

Tb content used for cation exchange (μmol)	Tb (ppm)	Yb (ppm)	Tb/Yb weight ratio	Tb/Yb molar ratio	Exchanged Tb^{3+} concentration in the $NaYF_4$ shell
0.2	2.45 ± 0.70	117.27 ± 22.22	2.1 %	1.93 %	> 3.86 %
0.4	3.82 ± 0.46	121.33 ± 18.90	3.1 %	2.85 %	> 5.69 %
1	4.75 ± 0.33	110.36 ± 14.60	4.3 %	3.95 %	> 7.90 %
2	6.14 ± 0.17	95.23 ± 2.10	6.4 %	5.88 %	> 11.75 %
4	8.43 ± 0.85	102.78 ± 10.33	8.2 %	7.53 %	> 15.06 %
8	10.19 ± 0.70	109.00 ± 5.00	9.3 %	8.54 %	> 17.08 %

Supplementary Note

Förster resonance energy transfer (FRET) efficiency strongly depends on the donor-acceptor distance[1-3]. It can be expressed by

$$\eta_{ET} = \frac{1}{1 + (\frac{r}{R_0})^6}$$

Where r is the distance between the donor and acceptor; R_0 is the Förster radius.

Förster Radius (R_0)

Förster radius R_0 is the donor–acceptor separation at which the FRET efficiency is 50%.

$$R_0 = \left[\frac{9000(ln10)\kappa^2\Phi_D J(\lambda)}{128\pi^5 N_A n^4}\right]^{1/6}$$

where Φ_D is the quantum yield of donor chromophore in the absence of acceptor, $J(\lambda)$ is the spectral overlap integral, κ^2 is the directional relationship of transition dipoles, n is the refractive index of the medium, and N_A is Avogadro's number.

Spectral Overlap

The overlap integral $J(\lambda)$ represents the extent of overlap between the donor emission and the acceptor absorption. The overlap integral is given by

$$J = \int_0^{\infty} F_D(\lambda)\epsilon_A(\lambda)\lambda^4 d\lambda$$

where F_D is the normalized emission intensity of donor, ϵ_A refers to the molar absorption coefficiency of the acceptor, and λ is the wavelength.

Orientation of transition dipoles

FRET efficiency is also affected by orientations of the emission transition dipole of the donor and the absorption dipole of the acceptor. κ^2 is a geometrical factor that accounts for the relative orientation of the two dipoles. The average κ^2 is 2/3 for an arbitrary orientation.

Supplementary References

1. Chen, G. et al. Efficient broadband upconversion of near-infrared light in dye-sensitized core/shell nanocrystals. *Adv. Optical Mater.* **4**, 1760–1766 (2016).
2. Ha, T. et al. Probing the interaction between two single molecules: fluorescence resonance energy transfer between a single donor and a single acceptor. *Proc. Natl. Acad. Sci. U. S. A.* **93**, 6264–6268 (1996).
3. Bennett, R. G. Radiationless intermolecular energy transfer. i. singlet→singlet transfer. *J. Chem. Phys.* **41**, 3037–3040 (1964).
